# Experimental and numerical investigation of the axial compressive behavior of GFRP-reinforced concrete walls under concentric and eccentric loading

**DOI:** 10.1038/s41598-026-52146-x

**Published:** 2026-05-18

**Authors:** Taha A. El-Sayed, Mohamed M. Ibrahim, Ali S. Shanour, Yasser Galal Eldin Mohamed Fahmy

**Affiliations:** 1 Civil Engineering Department, Faculty of Engineering at Shoubra, Benha University, Cairo, Egypt; 2 Reinforced Concrete Institute, Housing & Building National Research Center (HBRC), Cairo, Egypt

**Keywords:** Reinforced concrete walls, GFRP bars, Axial centric loading, Eccentric compression, ABAQUS, Nonlinear finite element analysis, Concrete Damaged Plasticity (CDP), Finite element validation, Concentric loading, Eccentric loading, Engineering, Materials science

## Abstract

This study investigates the axial compressive behavior of reinforced concrete (RC) walls reinforced with glass fiber–reinforced polymer (GFRP) bars under concentric and eccentric loading through a combined experimental and numerical approach. The six RC wall specimens with dimensions of 1000 × 800 × 150 mm were tested and divided into two groups. For Group G1 there were three specimens tested for concentric axial loading and for Group G2 three specimens were analyzed for eccentric axial loading. One control wall in each group was reinforced with conventional steel bars and the rest were reinforced with GFRP bars in vertical and horizontal directions. The experimental program studied first-cracking and ultimate loads, cracking behavior, stress–strain response, ductility ratios, energy absorption capacity, and lateral displacements. The results indicated that replacement of steel reinforcement with GFRP bars decreased ultimate axial capacity; however, GFRP-reinforced walls maintained stable post-cracking behavior and satisfactory ductility performance. When subjected to concentrically loaded specimens, the ultimate load capacity of GFRP-reinforced walls decreased by approximately 10.8–13.3% compared with the steel-reinforced control specimen, while the ductility ratio increased by about 4.4–4.8% points. Under eccentric loading, the ultimate capacity reduction ranged from 6.5% to 14.1% relative to the steel control wall. The energy absorption capacity, assessed from the load–displacement response, was lower for GFRP-reinforced walls compared to steel-reinforced specimens; however, a stable post-cracking response was maintained under both concentric and eccentric loading. In ABAQUS, nonlinear finite element models were generated through the Concrete Damaged Plasticity (CDP) model to emulate the structural response of the tested walls, to be complementary to the experimental study. The numerical results agreed well with the experimental results regarding ultimate capacity, load–deformation behavior, stiffness degradation, and crack distribution. The deviation between experimental and numerical ultimate loads ranged from approximately 0.25% to 11.9%, confirming the reliability and acceptable predictive accuracy of the adopted numerical modeling approach. Therefore, in general, in RC wall systems it is concluded that GFRP bars present a potential viable corrosion-resistant alternative to traditional steel reinforcement. The combined experimental and numerical results yield useful information about the axial behavior of GFRP-reinforced concrete walls under concentric and eccentric loading, that can be useful for making a better design decision in future for durable and sustainable structural wall applications.

## Introduction

 Reinforced concrete (RC) walls are fundamental structural components in modern construction, serving as both primary load-bearing members and lateral force resisting elements in high-rise buildings, bridge abutments, and infrastructures located in seismic regions^1,2^. Their role in resisting axial forces and contributing to global stability makes them indispensable in structural systems. However, despite their widespread use, RC walls remain vulnerable to premature deterioration and failure under extreme service demands, particularly due to corrosion of steel reinforcement and excessive load effects^3,4^. Corrosion of embedded steel bars not only reduces structural capacity but also significantly shortens the service life of RC structures, leading to considerable economic losses worldwide^5^.

To solve these limitations, fiber-reinforced polymer (FRP) composites have emerged as a promising alternative to typical steel reinforced systems. Among different classes of FRP, glass fiber–reinforced polymer (GFRP) bars have specifically attracted attention because of their high strength-to-weight ratio, corrosion resistance, and long-term durability^6–8^. Compared with steel, GFRP reinforcement is resistant to chloride-induced corrosion and alkaline attack, thus making it ideal for marine and chemically aggressive environments^9^. Moreover, its lightweight, non-magnetic aspect offers novel benefits for certain uses. However, GFRP has its disadvantages mainly its relatively low modulus of elasticity and brittle failure behavior, which cause greater displacements and larger crack widths in comparison to steel-reinforced materials^10,11^. These issues lead to a consideration of ductility and structural serviceability, but overall between durability and mechanical performance GFRP remains one of the most promising materials in sustainable concrete construction^12^.

GFRP bars are produced by continuous glass fibers in a polymeric resin matrix that provide the major tensile strength and transfer of stresses for good environmental protection. The typical manufacturing for GFRP bars is the pultrusion process, which is carried out in which glass fibers are impregnated with resin and pulled continuously through a heated die and give rise to bars with regular cross sections. This procedure assures good alignment of fibers, good quality and good mechanical properties. GFRP bars are often treated or deformed around the surface during manufacturing process to improve the bond with the concrete. Thus, the kind of fibers, resin composition and the manufacturing process exert a significant contribution on the properties of GFRP reinforcement^47,48^.

Over the past 20 years, a large body of literature has investigated the characteristics of RC elements reinforced with FRP bars. Experimental and analytical research on beams subjected to GFRP has taken to testing flexural strength, crack propagation, and deflection behavior and reported reduced ductility, stronger durability than in traditional beams^13,14^. Further study of shear behavior confirmed that application of GFRP stirrups provides improved shear resistance, with improvements depending on the detailing of 40%–80% based on strength^15^. Furthermore, hybrid reinforcement systems that utilize FRP in combination with steel or other synthetic fibers (e.g. polyvinyl alcohol (PVA)) have been designed for brittleness reduction and load redistribution, demonstrating high rates with better ductility and energy absorption^16^.

More recent studies abroad have shown significant improvements in the compressive properties of GFRP-reinforced concrete members and wall systems, reinforcing the international focus on corrosion-proofing material solutions^17–19^.

These advances reflect the flexibility provided by FRP reinforcement in both serviceability and strength for a host of structural members. More than for beams and slabs, recent research has been performed on columns and walls reinforced with FRP.

High-strength concrete (HSC) columns reinforced to the floor plates using basalt FRP (BFRP) showed competitive high compressive strength and high concentric strength durability^20^. Likewise, high-strength concrete slabs loaded with GFRP bars showed increased flexural stiffness and similar solid load-deflection behavior in those poured of high-strength concrete slabs with GFRP bars than steel reinforcement^21^. Besides, ferrocement-based composites are introduced as the alternative reinforcement in RC walls with good results regarding the crack characteristics, crack permeability and energy absorption during concentric and eccentric stress for RC walls^22^. In addition, high-performance geopolymer beams strengthening with GFRP bars showed significant advancement in stiffness and long-standing improvement and sustainability advantages, such as lower carbon dioxide emission^23^. These results highlight the increasing interest in FRP systems for enhancing the performance and service life of RC members. More recently, GFRP-reinforced compression members and hybrid FRP–steel systems have been evaluated for improved durability, ductility, and design issues associated with compression-based elements have been addressed^24–26^.

Despite these developments, the axial compressive behavior of RC walls reinforced with GFRP bars under concentric loading has not been well characterized. Most literature reviews have focused on flexural response, bond-slip relationships, and cyclic loading of FRP-reinforced beams and columns^27,28^. Thus, in contrast, comparatively few experimental or numerical studies have focused on the compressive performance and ductility of RC walls reinforced by GFRP^29,30^. The absence of specific research in this domain significantly hampers design codes and the practical usage of GFRP in wall elements. However, only a limited number of studies have specifically focused on GFRP-reinforced RC walls subjected to axial compressive loading, resulting in a notable gap in current design knowledge and code provisions.

.

While previous studies have investigated GFRP-reinforced walls under lateral or combined loading conditions, and others have applied CDP modeling to FRP-reinforced compression members, a comprehensive experimental–numerical investigation of GFRP-reinforced RC walls under concentric axial loading remains limited^31,32^.

Current ACI 440.1R and CSA S806 provisions are primarily developed for flexural members and provide limited guidance for GFRP-reinforced compression elements, particularly wall systems^33–35^. The contribution of GFRP bars in compression is fully neglected, potentially leading to conservative strength predictions and uncertain ductility considerations. Therefore, further experimental and analytical data are required to establish reliable design recommendations for GFRP-reinforced RC walls.The existing design provisions ACI 440.1R and CSA S806 and Eurocode 2 are mainly developed for conventional steel-reinforced concrete members or for FRP applications in flexural members. As a result, their use for GFRP-reinforced RC walls subjected to axial compression is limited. ACI 440.1R and CSA S806 overlook the contribution of GFRP reinforcement to compression, which could result in conservative estimates of axial capacity.

Eurocode provisions also fail to specifically address the behaviour of FRP-reinforced wall systems under axial loads. This lack of a proper consideration of the real characteristics of GFRP-reinforced RC walls implies the need for further experimental and analytical investigation. Aside from conventional RC components, the axial behavior of GFRP reinforced concrete columns and composite wall systems has been examined in existing research in recent years through experimental and numerical comparison techniques. These methods demonstrated that GFRP reinforcement contributes to the axial load capacity of compression members, although compared to steel with its lower elastic modulus, it is less effective. At the same time, the effect of concrete strength and wall shape on axial response of composite concrete–steel wall-based systems has been tested and the numerical simulations carried out have shown satisfactory agreement with experimental results^36,37^.

These results indicate an increasing attention to extending the application of GFRP reinforcement and advanced modeling to structural components dominated by compression. Yet, most of these studies have been on column members or composite arrangements; nevertheless, GFRP-reinforced RC walls subjected only to axial loading under its pure form have not been analyzed adequately. Despite the wide literature of FRP-reinforced concrete members, most of them have been concentrated on flexural response, bond performance and column responses when facing axial strains. On the other hand, the concentric and eccentric loading characteristics of GFRP-reinforced RC wall systems exhibit that the axial compressive effects they show are largely unexplored. In addition, few studies integrated experimental observations with validated nonlinear finite element modeling and direct evaluation of design code predictions with respect to such structural elements. Hence, continued efforts are required for a thorough exploration of experimental behavior, numerical modeling, and actual design assessment. Given this background, the following paper presents an experimental and numerical exploration of GFRP-reinforced RC walls subjected to axial loading, in combination with a critical assessment of existing analytical and code-based methodologies. This integrated framework contributes to the current understanding and thus to the development of increasingly powerful design recommendations for GFRP-reinforced wall systems.

The present study addresses this knowledge gap by experimentally and analytically investigating the compressive behavior of RC walls reinforced with GFRP bars under axial concentric loading. The study aims to evaluate ultimate load capacity, cracking patterns, stress–strain response, ductility index, and energy absorption, and to compare with those in traditional steel-reinforced walls. Experimental results are also confirmed and investigated by complementary nonlinear finite element analysis to gain general insights into the structural response. The results of this study are expected to play a role in the development of design guidelines and to facilitate the wider deployment of GFRP reinforcement in RC walls. In addition to GFRP reinforcement, recent research has investigated basalt FRP (BFRP) and hybrid FRP systems in concrete members, addressing both flexural and axial behavior of beams, slabs, and columns. These studies point to the potential of FRP-based reinforcement solutions beyond conventional applications^38–41^.

## Aims of research

The current study aims to achieve the following aims:

- Experimentally assess the axial compressive behavior of reinforced concrete (RC) walls reinforced with glass fiber–reinforced polymer (GFRP) bars under concentric loading conditions.
- To study the influence of GFRP reinforcement ratio on the ultimate load capacity, stiffness, cracking patterns, and ductility of RC walls.
- To study the stress–strain response and energy absorption characteristics of GFRP-reinforced walls in comparison with conventional steel-reinforced counterparts.
- To validate the experimental findings through nonlinear finite element modeling, providing further insights into the mechanical response and failure mechanisms.
- To evaluate the advantages and possible limitations of GFRP reinforcement in RC walls and offer recommendations for future applications and design considerations.
- The study critically assesses current design provisions (ACI 440.1R, CSA S806, and Eurocode 2), highlighting their limitations, and offers practical guidance for the design of GFRP-reinforced walls in aggressive environments.

## Experimental work

### Materials

1.Cement.

All specimens were made of an ordinary Portland Cement (OPC) known as CEM I 42.5 N as its main material for binding. This is equivalent to a 28-day compressive strength category (of 42.5 MPa) with normal early strength development and is widely used in structural concrete. Material properties of the cement were confirmed by Egyptian Standard Specifications ES 4756-1/2013 and Egyptian Code of Practice (ECP 203–2020)^42^. Important parameters were fineness, setting times, specific gravity, and compressive strength.

2. Fine and Coarse Aggregates.

Fine and coarse aggregates of natural siliceous sand with a fineness modulus of 2.65 and crushed dolomite with a maximum nominal size of 12.5 mm were used, which offered appropriate grading, low absorption, and no deleterious substances.

3. Silica Fume.

Silica fume was selected as the supplementary cementitious material with 10% replacement by weight of cement, complying with ECP 203/2020 and ASTM C1240^44^. It is mainly made from amorphous SiO₂ (> 90%) and, due to its ultrafine size and pozzolanic activity, it enhances strength, reduces permeability, and improves durability as shown in Table 1.

**Table 1:** Chemical composition and physical properties of silica fume.

Property	Value
Sion₂ content (%)	96.5
Specific gravity	2.2
Surface area (m²/kg)	17,500
Bulk density (kg/m³)	200
L.O.I (%)	1.16

4. Mixing Water.

Potable tap water free from salts and impurities was used for both mixing and curing.

5. Superplasticizer.

A polycarboxylate-based high-range water-reducing admixture was used to maintain workability without increasing the water-to-binder ratio. The effective technical properties are presented in Table 2.

**Table 2:** properties of the superplasticizer.

Property	Value/Description
Density at 25 °C (kg/L)	1.18 ± 0.01
Actual dosage used	3.13% of cement weight

6. Steel Reinforcement.

High-strength deformed steel bars of Grade 60 were used in the control wall specimens. This grade corresponds to a nominal yield strength of 420 MPa and an ultimate strength of approximately 600 MPa, in accordance with the Egyptian Code of Practice (ECP 203–2020)^42^.

Two diameters were used: Ø10 mm for horizontal reinforcement in selected specimens and Ø12 mm for vertical reinforcement in others. These reinforcements served as a benchmark for comparison with GFRP-reinforced specimens.

7. GFRP Bars.

Glass Fiber Reinforced Polymer (GFRP) bars supplied by a certified manufacturer were tested according to ASTM D7205^43^. Their mechanical properties are listed in Table 3. Furthermore, the actual bar diameters were measured using a digital caliper and were found to be consistent with the nominal values. It should be noted that the reported mechanical properties correspond to straight GFRP bars obtained from standard tensile testing, whereas the bent portions shown in Fig. 2 represent reinforcement detailing and may exhibit different local behavior.

**Table 3:** Mechanical properties of GFRP reinforcement bars.

Property	Value
Ultimate tensile strength	1200 MPa
Elastic modulus	≈ 50 GPa
Stress–strain response	Linear to failure
Key advantages	Non-corrosive, lightweight, durable

### RC walls preparation

The experimental program included testing of six reinforced concrete wall specimens. All samples were constructed with a high-performance concrete (HPC) mix with a target cube compressive strength of 60 MPa, as presented in Table 4.

**Table 4:** Concrete mix proportions for wall specimens.

Materials	Quantity (kg/m³ or L/m³)
Cement	575 kg/m³
Silica fume	50 kg/m³
Coarse aggregate	1100 kg/m³
Fine aggregate	580 kg/m³
Water	138 kg/m³
Superplasticizer	18 L/m³
Target strength fcu	60 MPa

The wall specimens measured 1000 mm in height, 800 mm in length and 150 mm in thickness. The wall slenderness ratio was considered as height-to-thickness ratio (h/t) where h = 1000 mm and t = 150 mm, and.

h/t = 6.67. This measurement value is below the accepted threshold (≈ 10 for slender behavior), and the specimens studied are therefore categorized as short/stocky walls, having almost no global buckling susceptibility under concentric axial compression. The plan aspect ratio, the wall height-to-length ratio (hw/lw), is consistently equal to 1.25 as we find hw = 1000 mm and lw = 800 mm. This also locates the specimens in the squat range where axial–shear interaction dominates, rather than slender flexural instability. The fixed boundary conditions at both ends of the top and the bottom also diminish the effective slenderness, which endorses such a classification.

The specimens were divided into two main groups according to the type of axial load applied: concentric loading (Group G1) and eccentric loading (Group G2). Each group included one control specimen reinforced with conventional steel bars and two specimens reinforced with Glass Fiber Reinforced Polymer (GFRP) bars. The reinforcement configuration for all walls consisted of 6Ø12/m vertical reinforcement and either 5Ø10/m or 7Ø10/m horizontal reinforcement, as summarized in Table 5.

Group G1 specimens were tested under concentric axial loading to investigate the comparative behavior of steel- and GFRP-reinforced walls under uniform compression. Group G2 specimens were subjected to eccentric axial loading to evaluate the influence of reinforcement type and horizontal reinforcement ratio on the load-carrying capacity, cracking response, and deformation characteristics. All specimens were designed as under-reinforced sections to ensure that failure occurred primarily in the concrete or reinforcement system.

The vertical reinforcement was identical for all wall specimens (6Ø12/m), yielding ρv = 0.452%. The horizontal reinforcement varied among walls: specimens S1, S1-1, S2 and S2-1 used 5Ø10/m (ρh = 0.262%), whereas S1-2 and S2-2 used 7Ø10/m (ρh = 0.367%). These reinforcement ratios were adopted in the analytical calculations and FE modeling to ensure consistency between experimental and numerical behavior.

The selection of the investigated parameters was intended to focus on the influence of reinforcement type and loading condition, while maintaining other key variables constant. In particular, the concrete compressive strength and vertical reinforcement ratio were kept the same for all specimens to ensure a consistent basis for comparison and to isolate the effect of the selected variables on the structural response of the walls.

**Table 5:** Details of tested wall specimens.

Specimen group	Specimen symbol	Dimensions (cm)	Type of load	Vertical RFT type	Horizontal RFT type	Reinforcement (Ver. & Hzl.)	Reinforcement ratios	
							ρv (%)	ρh (%)
**G1**	S1	100 × 80 × 15	Concentric	Steel	Steel	6Ø12/m; 5Ø10/m	0.452	0.262
	S1-1			GFRP	GFRP	6Ø12/m; 5Ø10/m	0.452	0.262
	S1-2					6Ø12/m; 7Ø10/m	0.452	0.367
**G2**	S2		Eccentric	Steel	Steel	6Ø12/m; 5Ø10/m	0.452	0.262
	S2-1			GFRP	GFRP	6Ø12/m; 5Ø10/m	0.452	0.262
	S2-2					6Ø12/m; 7Ø10/m	0.452	0.367

### Specimen preparation and casting

**Fig. 1:**
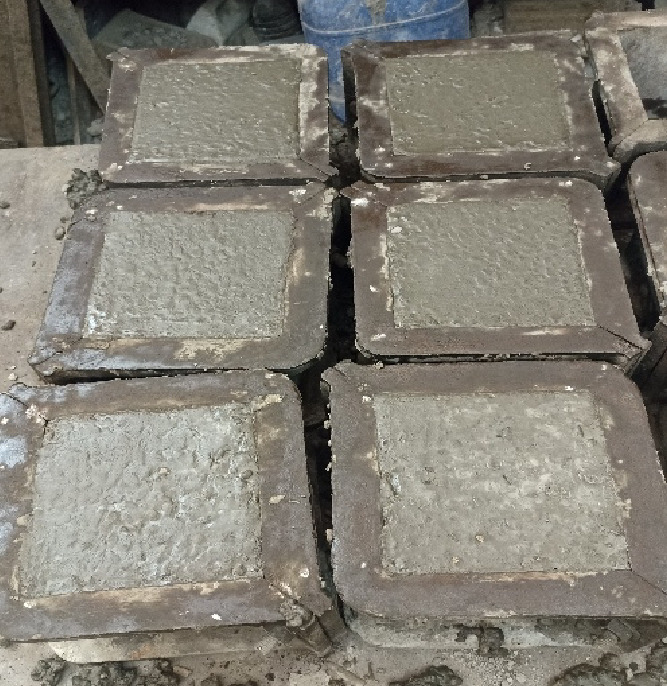
Concrete cube specimens prepared for compressive strength testing.

**Fig. 2:**
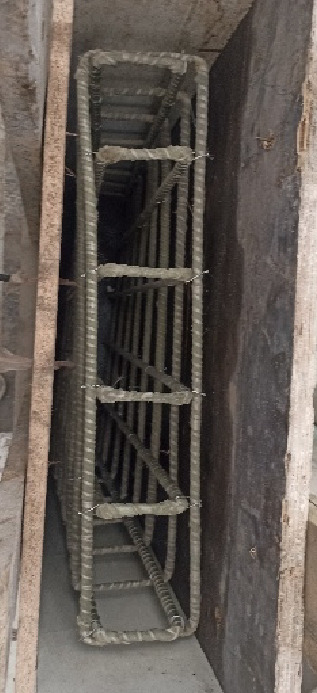
Reinforcement cage placed inside the wooden formwork of the wall specimen.

**Fig. 3:**
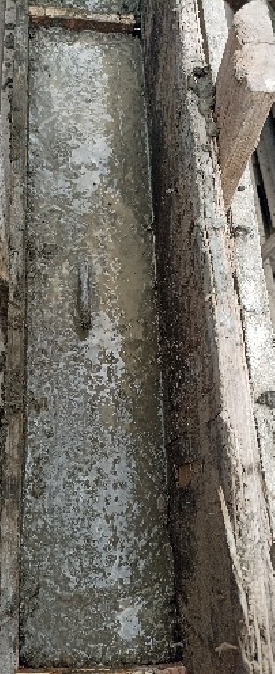
Casting of the concrete wall specimen in the prepared formwork.

Reinforced concrete wall specimens were prepared and cast under controlled laboratory conditions, as in Fig. 1. To prepare compressive strength test samples, standard cube molds were filled with the concrete mix to guarantee the quality and consistency of the actual concrete mixtures utilized in full-scale specimens. Reinforcement cages of GFRP bars with diameters of 10 mm and 12 mm were prepared and laid in the wooden molds shown in Fig. 2. The cages were adjusted to the proper alignment and fixation to ensure the reinforcement was correctly placed when casting. The concrete was then poured into the molds in layers, with each layer adequately compacted to eliminate air voids and ensure uniform distribution. Surface finishing was done to achieve a uniform top level, as shown in Fig. 3. All specimens were cured under standard conditions before testing in order to obtain sufficient hydration and strength development.

### Compressive strength of concrete

**Fig. 4:**
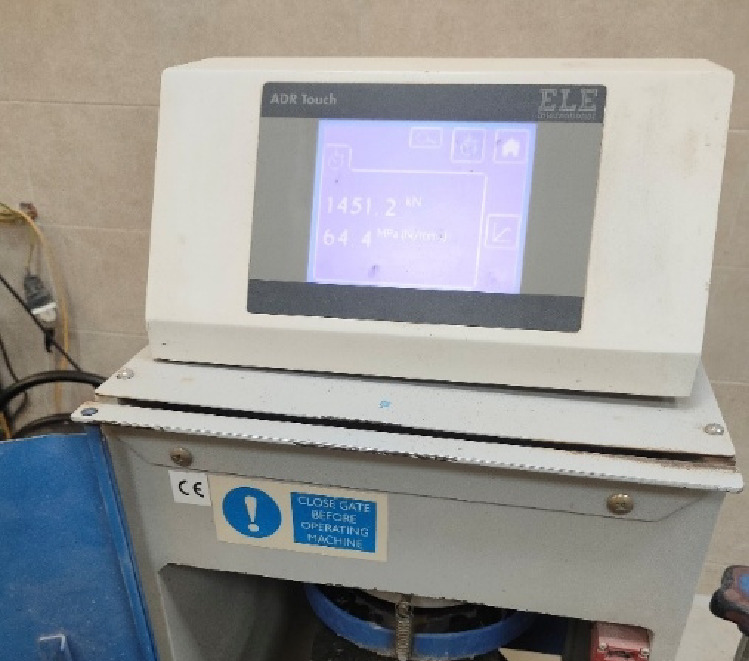
Concrete cube specimen after compressive strength test (64.4 MPa).

The compressive strength of concrete is determined by applying tests on standard cube specimens with dimension 150 × 150 × 150 mm at 28 days as provided in Fig. 4 based on the Egyptian Code of Practice (ECP 203). Results were compiled as shown in Table 6. However, cylinder specimens are more widely used in many international standards, although cube specimens are most frequently found in Egyptian practice. The corresponding mechanical properties of concrete were estimated based on the obtained compressive strength and established commonly accepted empirical relationships. The modulus of elasticity (Ec) calculated as Ec = 4400√fcu yielding a value around 34.1 GPa. The tensile strength (ft) was expressed as ft ≈ 0.6√fcu, while the modulus of rupture (fr) was defined as fr ≈ 0.7√fcu. These expressions are extensively used, particularly for high-performance concrete, and were chosen in this study to interpret experimental results and to keep up with finite element modelling.

**Table 6:** Compressive strength test results of concrete cubes.

Cube	Compressive Strength (MPa)
C1	64.4
C2	60.4
C3	62.7
Avg.	62.5

### Specimen preparation and test setup

**Fig. 5:**
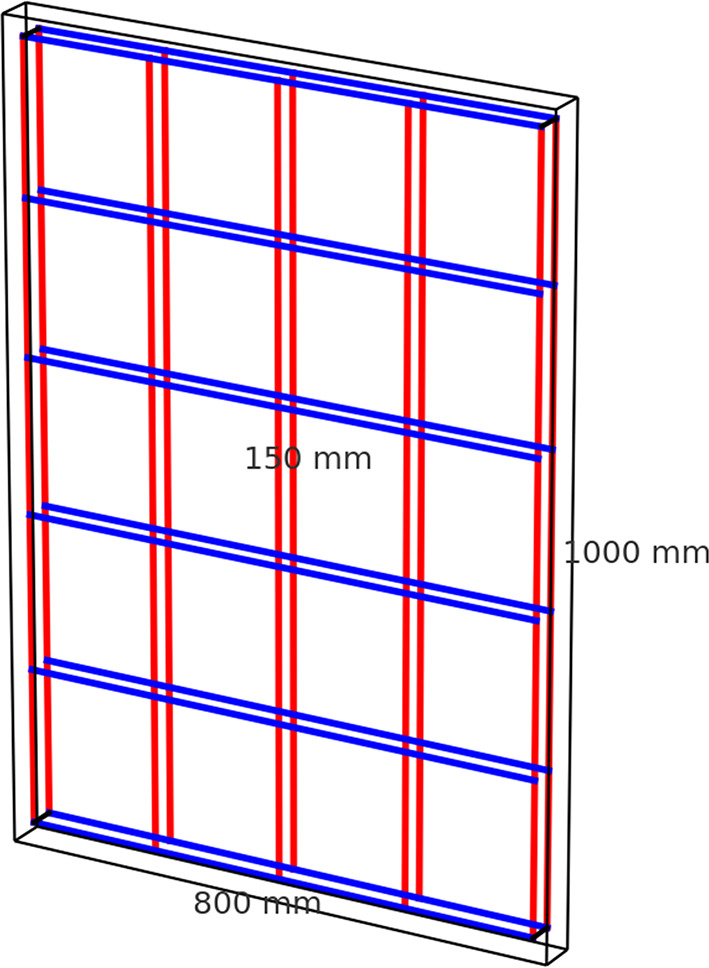
Geometry and reinforcement details.

All reinforced concrete wall specimens were of 1000 mm in height, 800 mm long, and 150 mm thick. The reinforcement was in closed-loop vertical and horizontal bars in mesh geometry to have a uniform distribution and sufficient confinement behavior. Reinforcement configuration and specimen shapes are shown in Fig. 5.

**Fig. 6:**
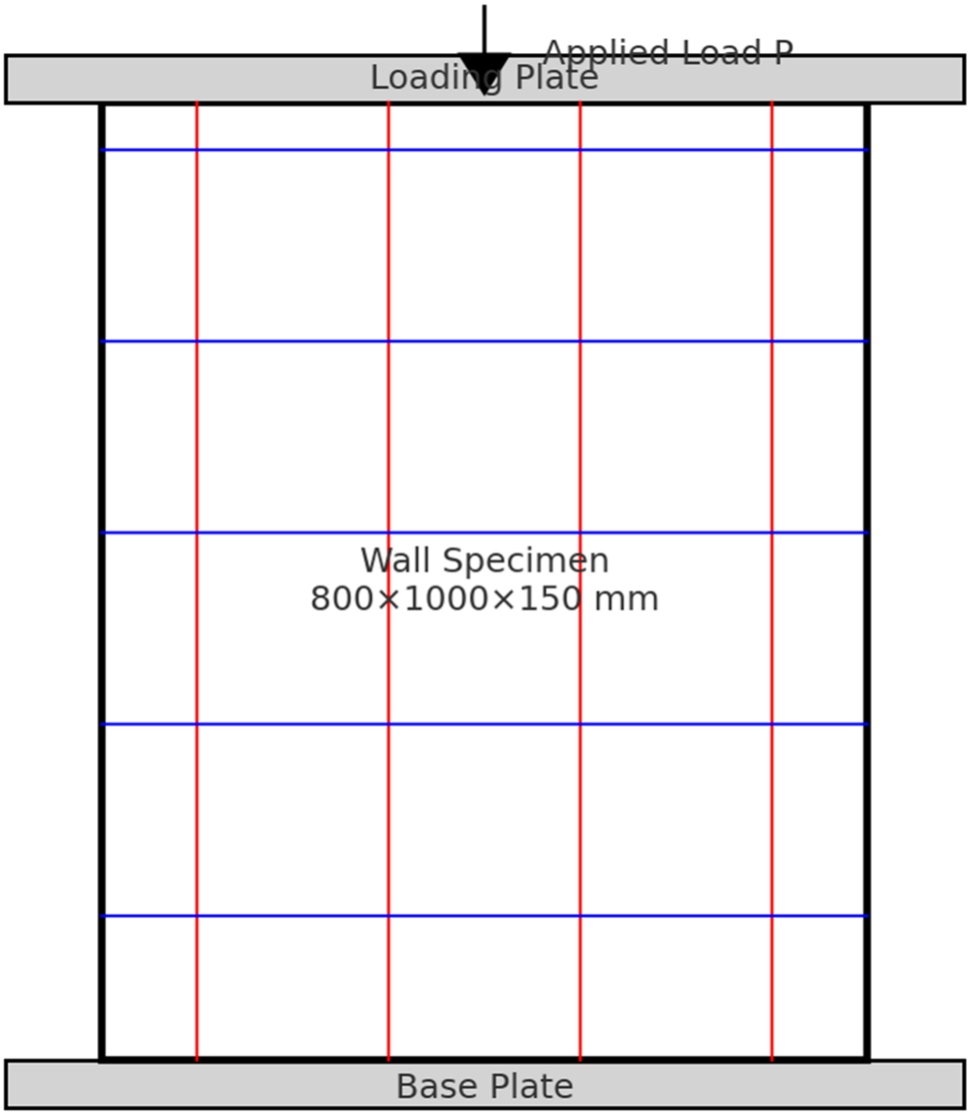
Test setup of the wall specimen under axial loading.

For testing, each wall specimen was placed in a universal testing machine and loaded axially under either concentric or eccentric conditions, depending on the designated test group. The load was applied through a rigid steel loading plate at the top, while the bottom surface was supported on a fixed steel base plate to ensure uniform load transfer. The applied axial load (P) was gradually increased until failure, as schematically shown in Fig. 6.

All experimental tests were conducted at the Housing and Building National Research Center (HBNRC), Dokki, Egypt, using a 5000 kN capacity testing machine.

of the tested wall specimen.

### Test setup and instrumentation

**Fig. 7:**
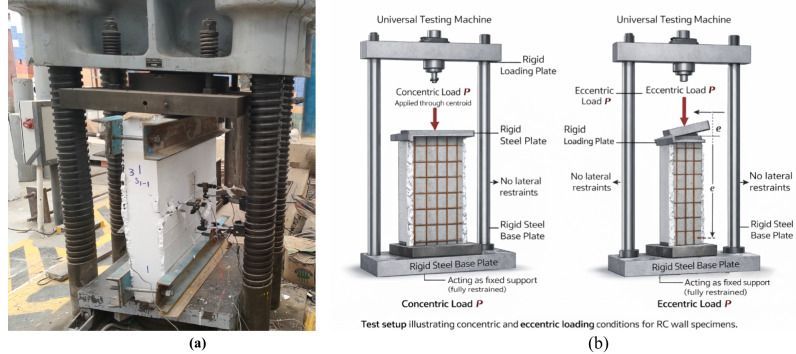
Test setup of wall specimens: (**a**) general view of the experimental setup; (**b**) schematic illustration of loading system, support conditions, and eccentric loading configuration.

Testing of all wall specimens was carried out using a universal testing machine with a maximum capacity of 5000 kN. Each specimen was positioned vertically between rigid steel loading and base plates to ensure uniform transfer of the applied axial load, as illustrated in Fig. 7(a), while the corresponding boundary conditions and loading configurations are shown schematically in Fig. 7(b). The load was applied concentrically or eccentrically depending on the test group.

The support conditions were defined to ensure stable and consistent load transfer during testing. Each wall specimen was placed on a rigid steel base plate that acted as a fixed support. The applied load was transferred through a rigid steel loading plate positioned at the top of the specimen to provide uniform stress distribution.

For concentrically loaded specimens, the load was applied through the centroid of the wall cross-section. The necessary eccentricity that was applied for the eccentric loading was obtained by moving the actuator’s position so that the line of action of the load did not pass through the centroid of the specimen as illustrated in Fig. 7(b). No lateral restraints were provided along the height of the specimen, allowing the wall to deform freely under the applied load.

**Fig. 8:**
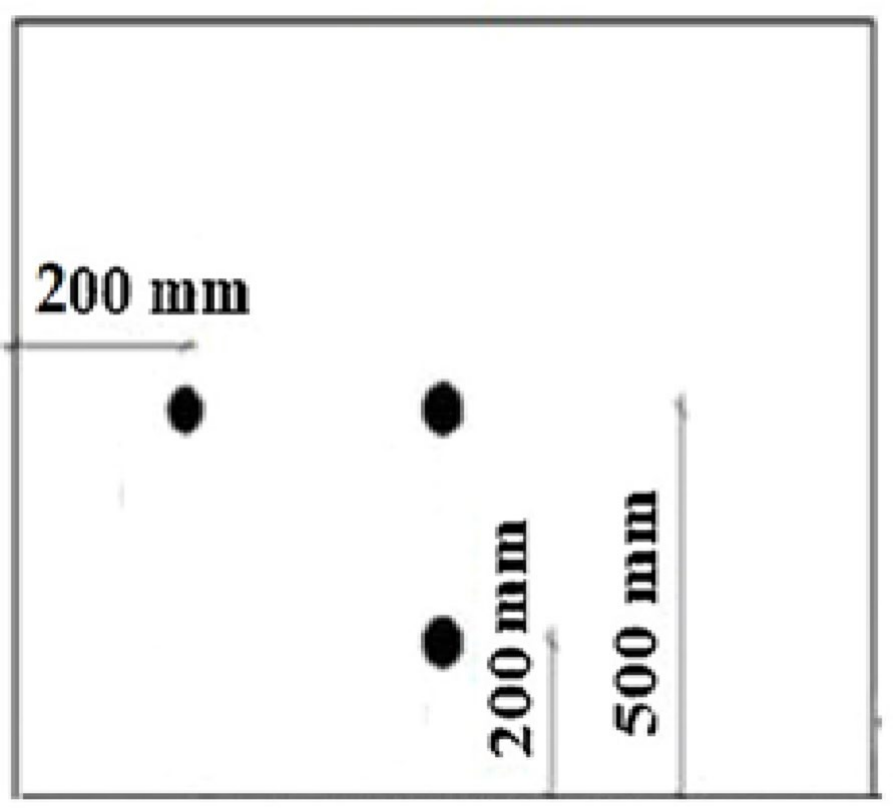
LVDTs Location on the tested wall specimens.

The response of the specimens was evaluated by fitting electrical resistance strain gauges to one vertical and one horizontal reinforcement bar for recording of reinforcement strains under loading. Linear Variable Differential Transformers (LVDTs) were also applied at selected points in order to measure axial displacement until failure, as shown in Fig. 8.

Therefore, the instrumentation had been designed with a high resolution and calibration accuracy, leading to meaningful measurements. The displacement measurement, based on LVDTs that had a sensitivity of 1 μm and full-scale stroke of 25 mm, could be made to notice small deformation changes before the cracks occurred and before the maximum load. All strain gauges had 10 mm gauge length and nominal resistance of 120.4 Ω ± 0.4% (KYOWA KFSG-10-120-C1-11L2M2R, Japan), yielding ± 0.5% precision measurement. All LVDTs were aligned parallel with the applied deformation axis and fully zeroed ahead of the loading. The sensors were all calibrated prior to any operation, and the data acquisition unit sampled at a frequency of 10 Hz to obtain consistent readings during the testing period.

**Bond Considerations**.

While no pull-out bond test was performed as a characteristic of this While no dedicated pull-out bond test was conducted within the scope of this experimental program, the effect of GFRP-to-concrete bond was inherently captured through the measured global load–displacement response of the full-scale wall specimens. Previous studies have demonstrated that GFRP–concrete bond has a significant influence on axial stiffness and post-peak response of FRP-reinforced compression members. Accordingly, the present interpretation and FE calibration relied on the actual measured structural response of the specimens rather than assuming isolated bond coefficients from small-scale pull-out tests.

#### Loading technique

**Fig. 9:**
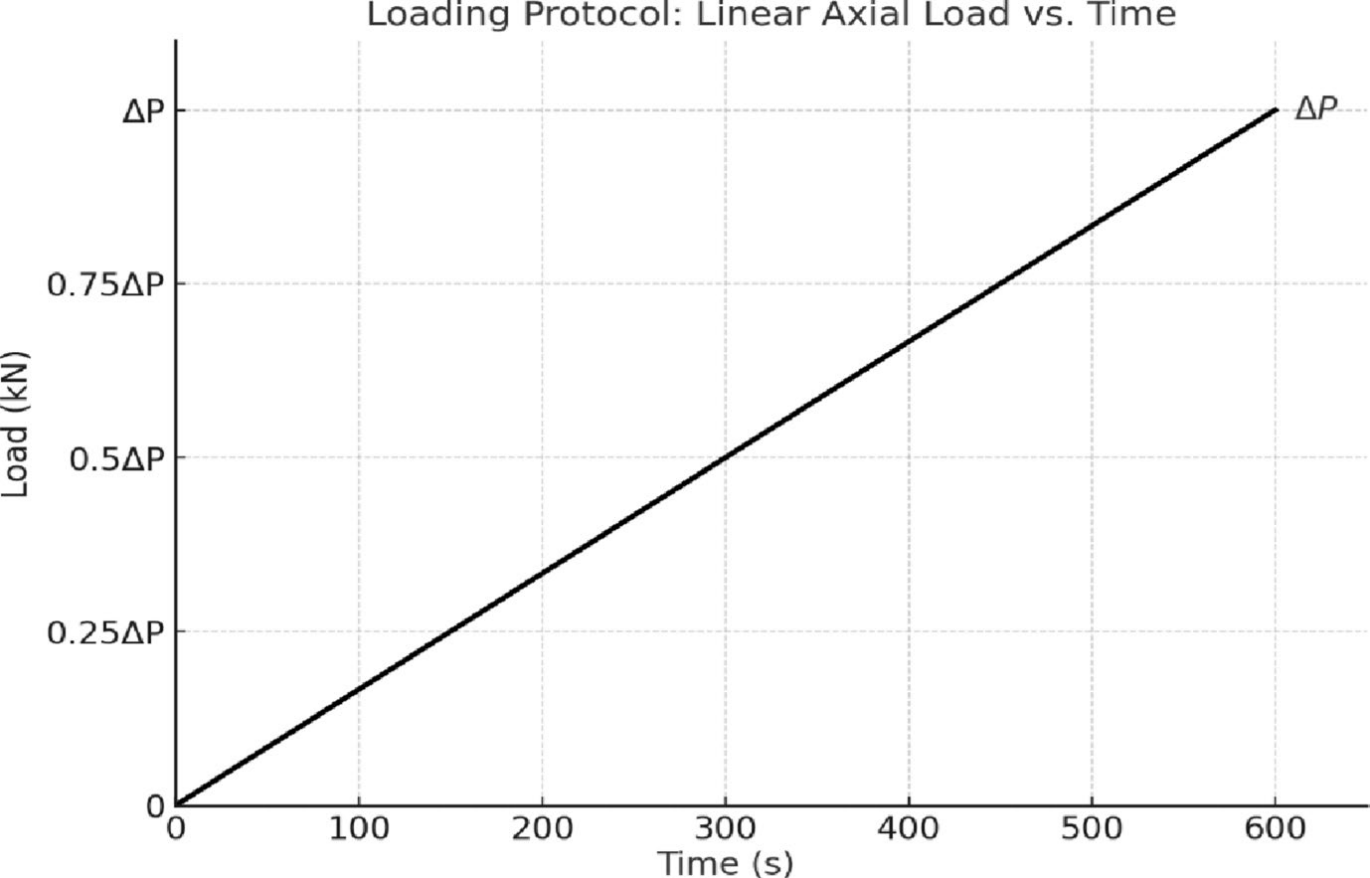
Loading protocol: linear increase of axial load with time (ΔP).

**Fig. 10:**
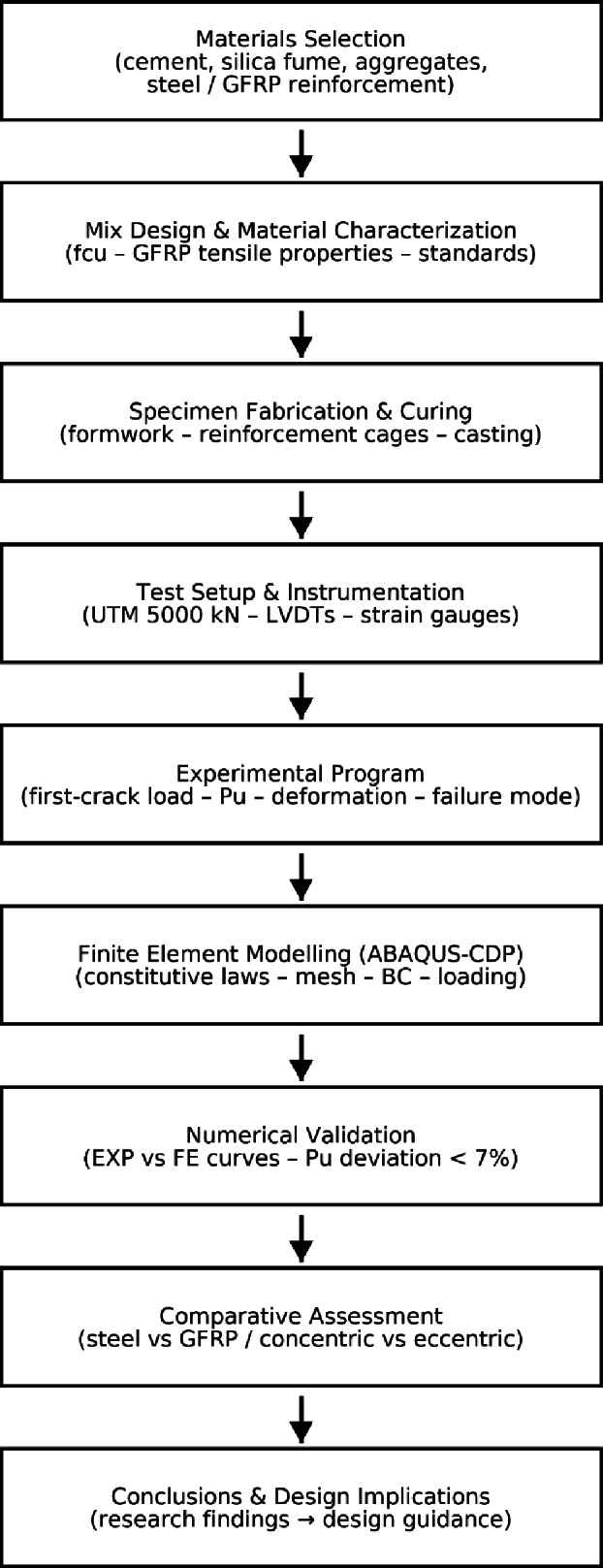
Flowchart illustrating the complete methodology of the present study (materials → experimental testing → numerical modeling → validation → conclusions). Abbreviations: UTM (Universal Testing Machine), LVDT (Linear Variable Differential Transformer), EXP (Experimental), FE (Finite Element), and BC (Boundary Conditions).

The loading procedure applied in the current study was through the application of a monotonic axial compressive load with displacement control. The load was applied at a constant rate of 0.5 mm/min and increased linearly with time until the specimen reached its ultimate capacity (ΔP). The loading scheme used is reported in Fig. 9. The applied load eccentricity (e) was obtained with regard to the wall thickness (t). For the above study, an eccentricity of e = 37.5 mm was chosen, representing an eccentricity ratio of e/t = 0.25 (i.e., e = t/4 for wall thickness of 150 mm). Such a number had been chosen to show a noticeable bending effect and to take into account the simultaneous axial and flexural loading of the wall specimens which were examined (Fig. 10).

### Crack patterns and final failure modes

**Fig. 11:**
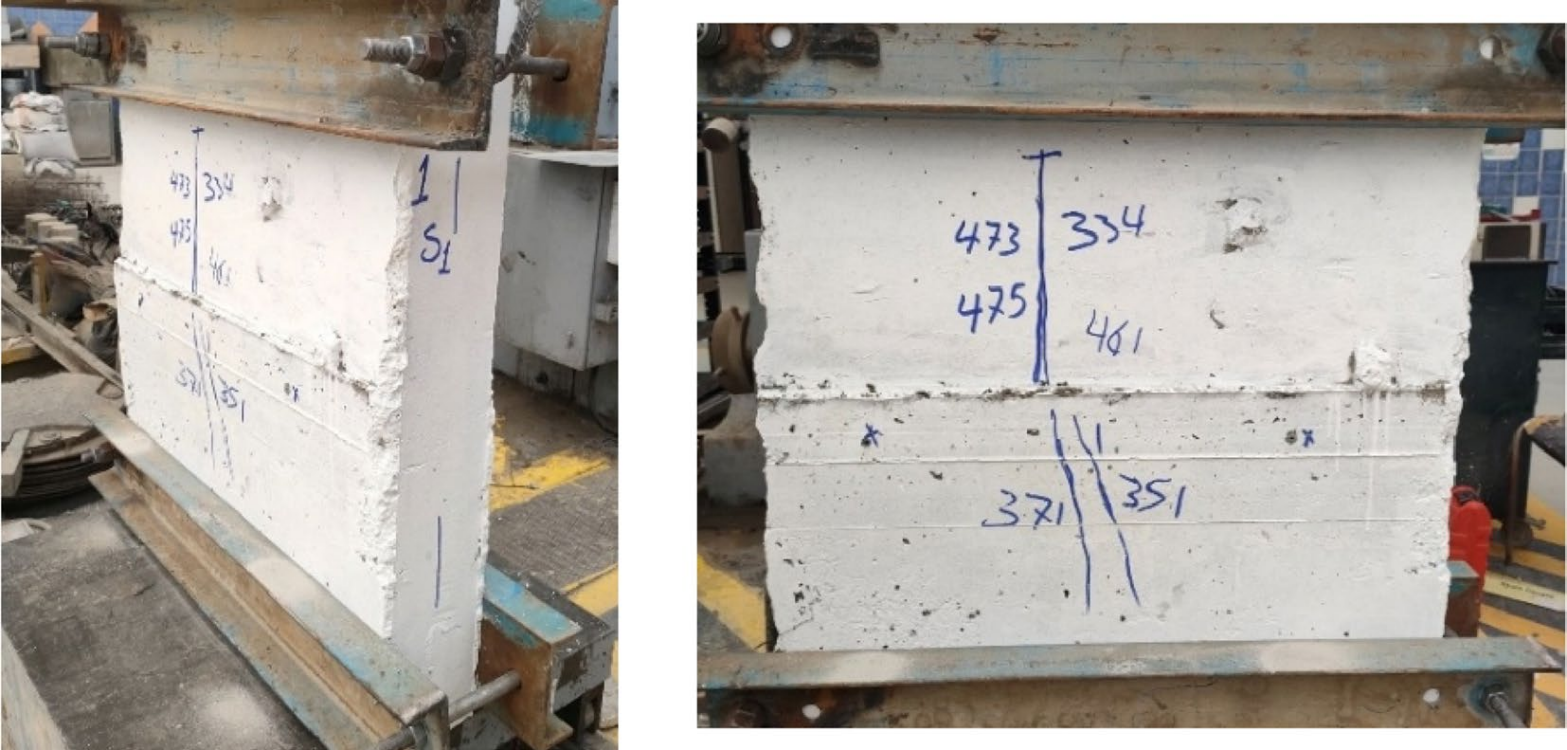
Typical crack pattern of wall specimen S1 under concentric axial load.

**Fig. 12:**
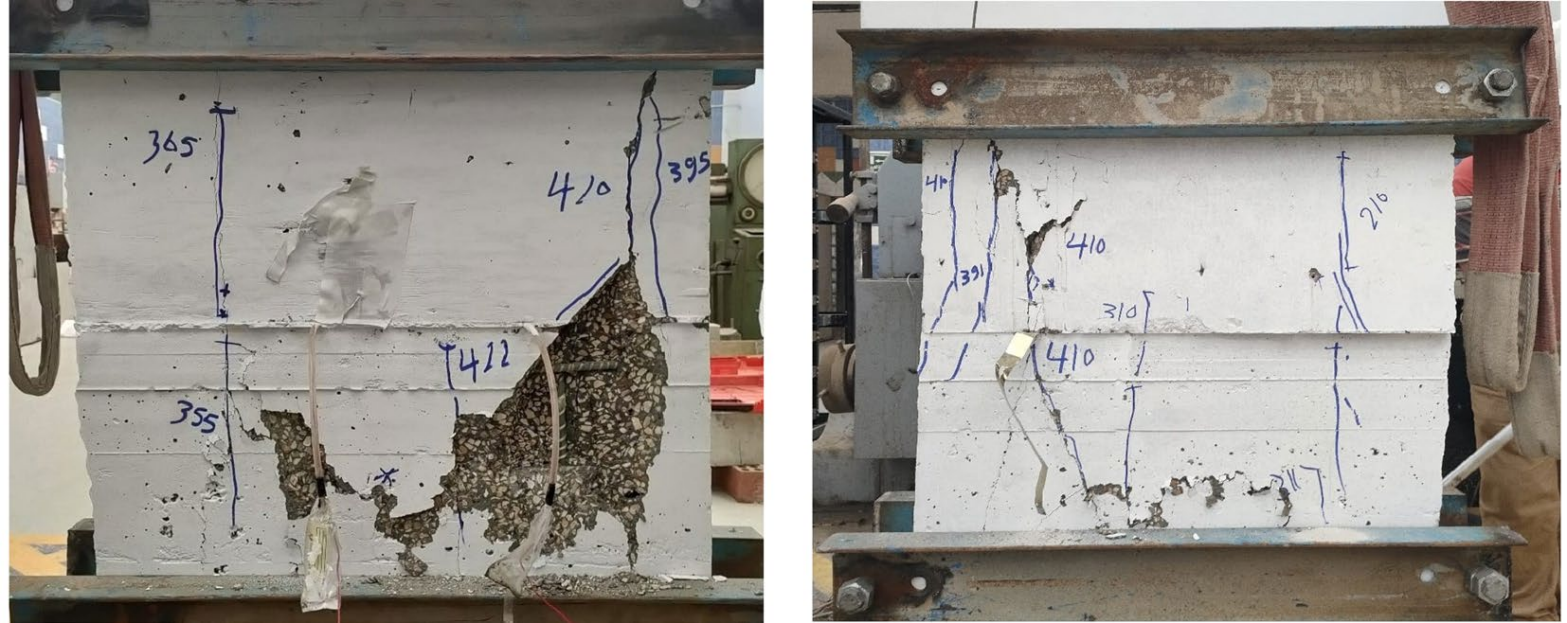
Typical crack pattern of wall specimen S1-1 under concentric axial load.

**Fig. 13:**
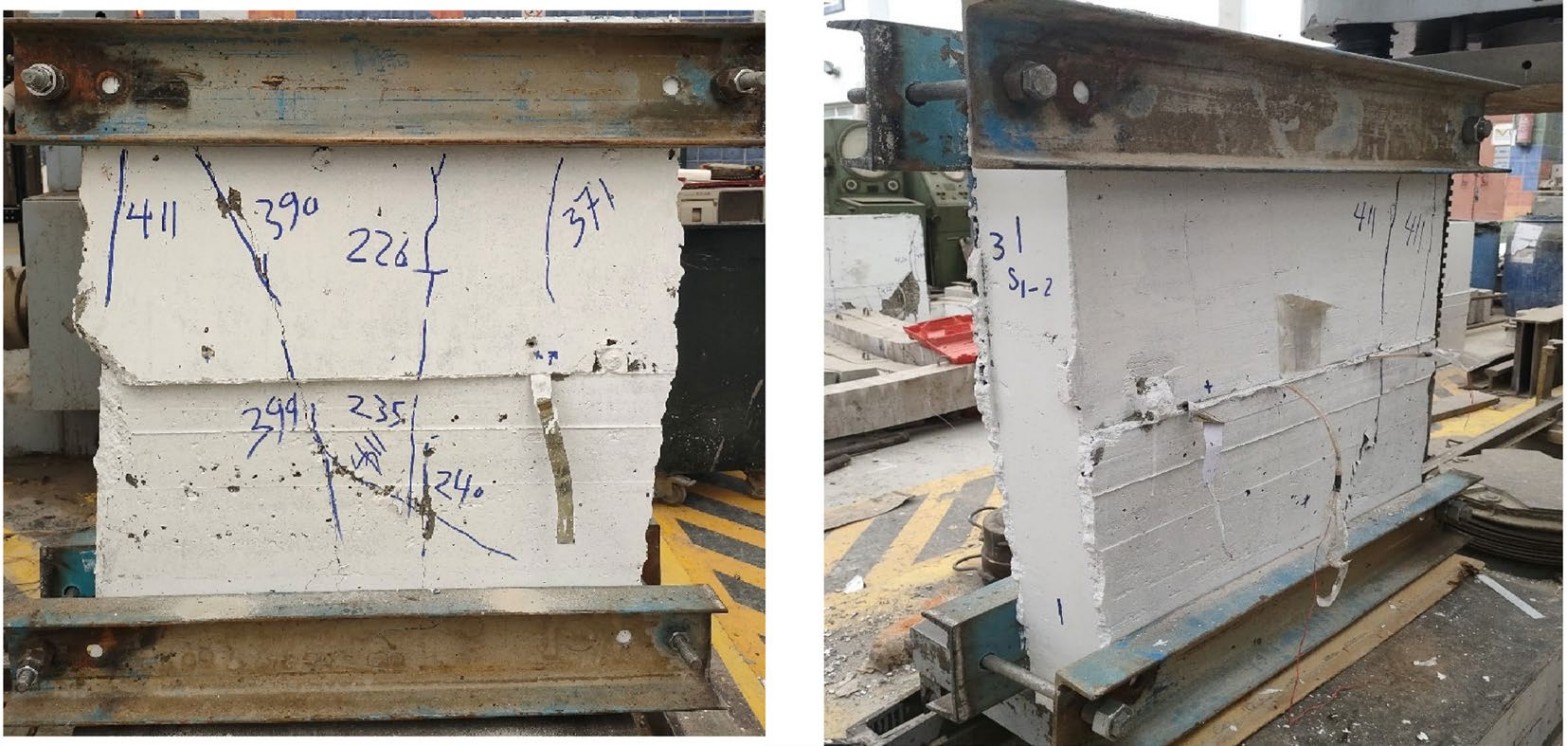
Typical crack pattern of wall specimen S1-2 under concentric axial load.

**Fig. 14:**
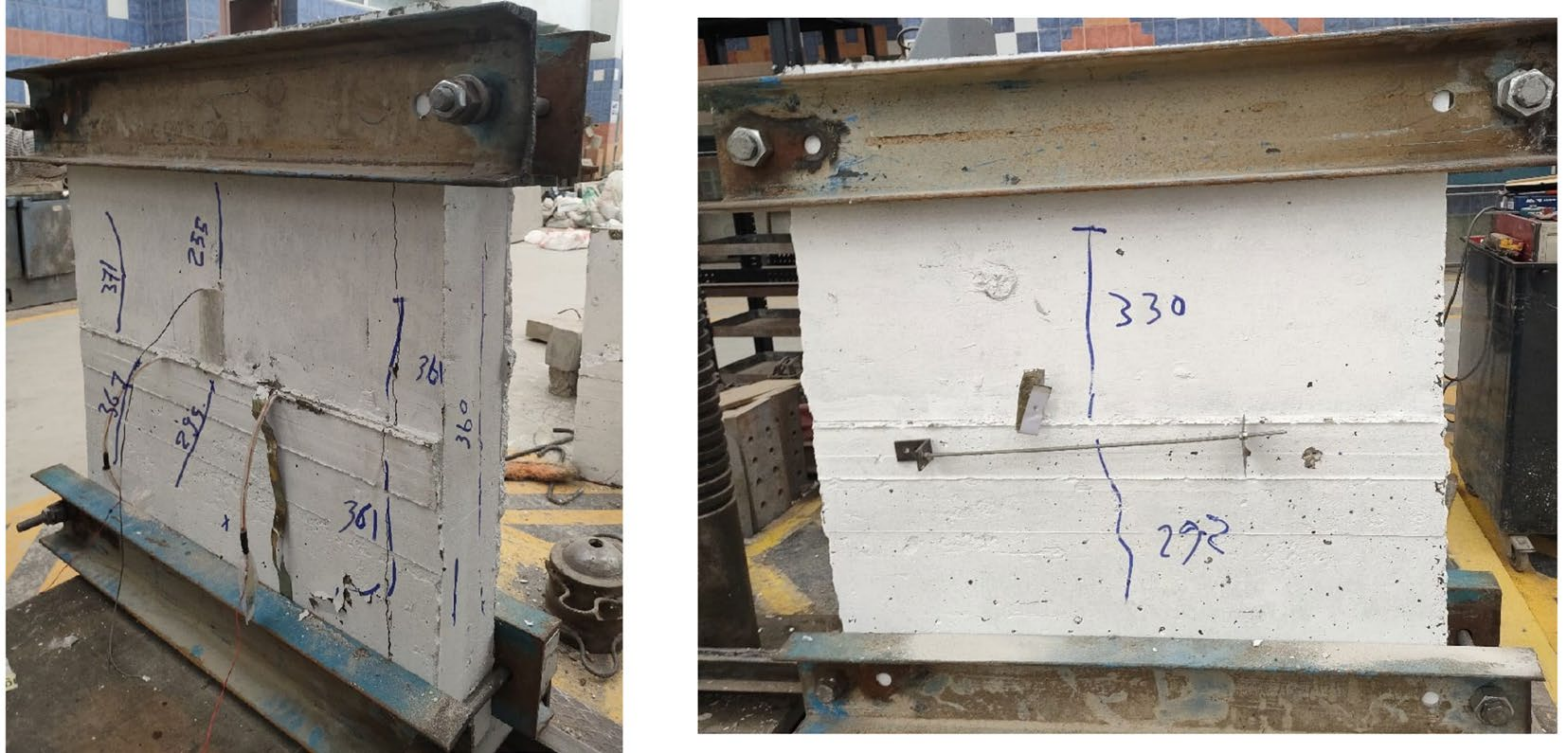
Crack pattern of wall specimen S2 under eccentric axial load.

**Fig. 15:**
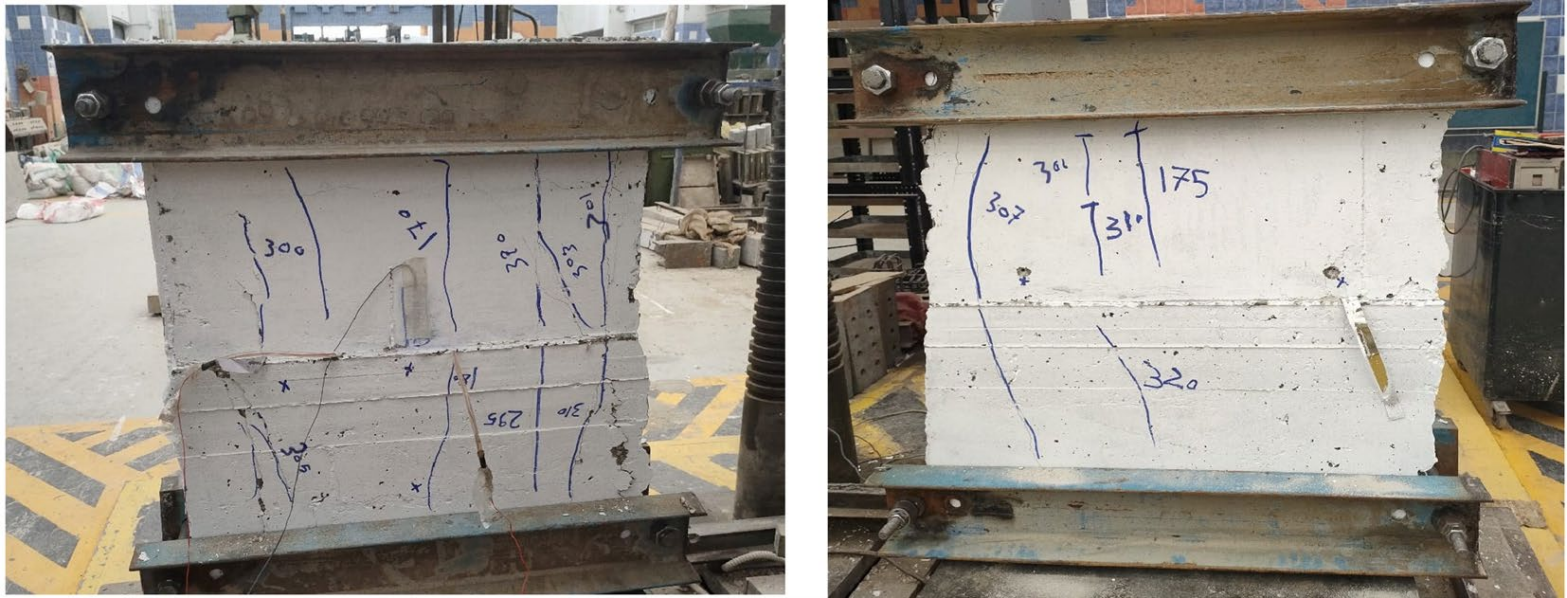
Crack pattern of wall specimen S2-1 under eccentric axial load.

**Fig. 16:**
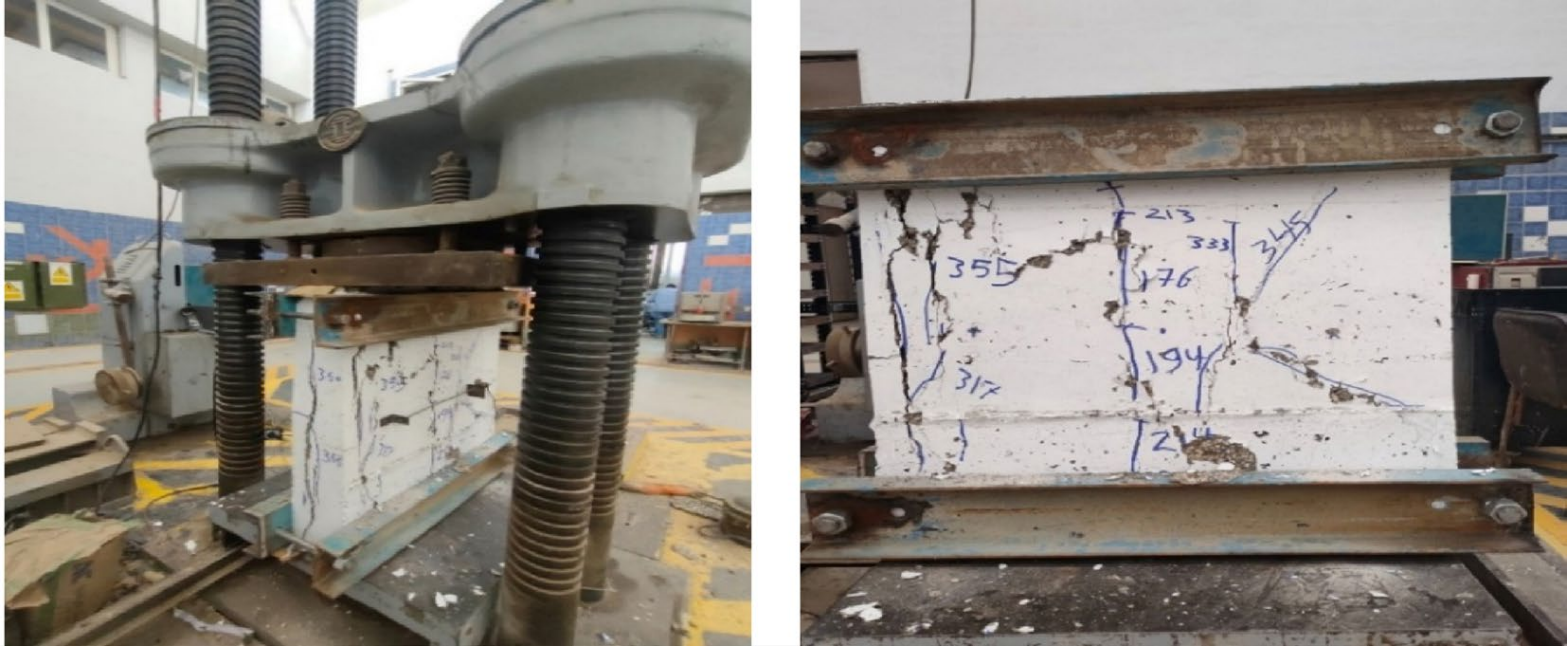
Crack pattern of wall specimen S2-2 under eccentric axial load.

Figures below show the crack patterns detected in the studied wall specimens. Vertical cracks were initially observed close to the mid-height and they continued to spread as the load increased. In specimens subjected to eccentric loading, inclined cracks formed early on the compression side and projected toward the tension zone. At higher load levels, crack widths increased and spalling of the concrete cover was observed in the compression region. The failure observations at the end revealed a significant difference in reinforcement types. Steel-reinforced walls mainly collapsed due to severe concrete crushing and cover spalling at the compression toe, followed by gradual failure after steel yielding. GFRP-reinforced walls, however, exhibited more distributed cracking with limited spalling, and failure was abrupt because of peak load induced GFRP bar rupture. This indicates a more ductile post-peak behavior for steel walls, and a more brittle collapse mode for GFRP walls under axial compression (Figs. 11, 12, 13, 14, 15, 16).

### Test results of wall specimens

The first crack and ultimate load figures in the reported data reflect the response of each wall specimen subjected to various reinforcement configurations and loading profiles. These results are shown together to emphasize the dependence of reinforcement type and load eccentricity on the axial behavior of RC walls. The test results are summarized in Table 7.

**Table 7:** Test results of wall specimens.

Specimen symbol	load test Type	First crack load (kN)	Failure load (kN)
**S1**	Concentric	3340	4751
**S1-1**		2100	4119
**S1-2**		2260	4237
**S2**	Eccentric	2550	3748
**S2-1**		1700	3221
**S2-2**		1940	3504

The results presented in Table 7 have been reviewed as to the relevant design standards. The crack initiation limits for crack initiation in GFRP-reinforced walls are not explicitly stipulated in prevailing codes, but at present the observed failure loads are in the acceptable range in comparison with code-based predictions, as reported below in Table 9. This indicates that the tested specimens meet the required strength criteria, with a degree of conservatism evident in the design estimates.

**Fig. 17:**
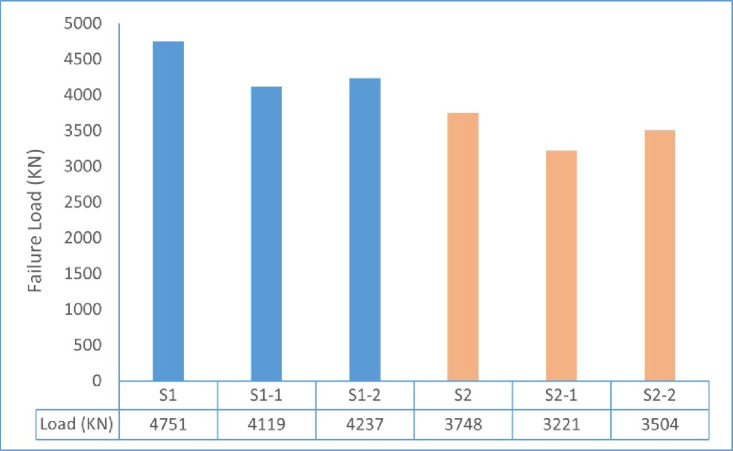
Experimental failure loads of tested walls.

Individual units of specimens tested under concentric loading (Group G1) showed higher failure loads compared to those tested under eccentric loading (Group G2) as shown in Fig. 17. The increase in load eccentricity is responsible for the reduction of the capacity under an eccentric response, resulting in the additional bending moment and an earlier crack propagation, reducing axial load resistance in the specimen. Moreover, failure loads compared to steel reinforced specimens have been slightly less for GFRP reinforcement, which are found to be due to lower modulus of elasticity of GFRP bars.

#### Ductility ratio

Ductility ratio of the tested wall specimens was evaluated based on the displacement response and defined as the ratio between the axial displacement at ultimate load (Δu) and the displacement corresponding to 0.8 of the ultimate load (Δ0.8Pu). Accordingly, the ductility ratio (µΔ) is expressed as: µΔ = Δu /Δ0.8Pu This definition provides a clear measure of the deformation capacity of the wall specimens beyond the elastic stage and allows consistent comparison between different reinforcement configurations and loading conditions.

**Fig. 18:**
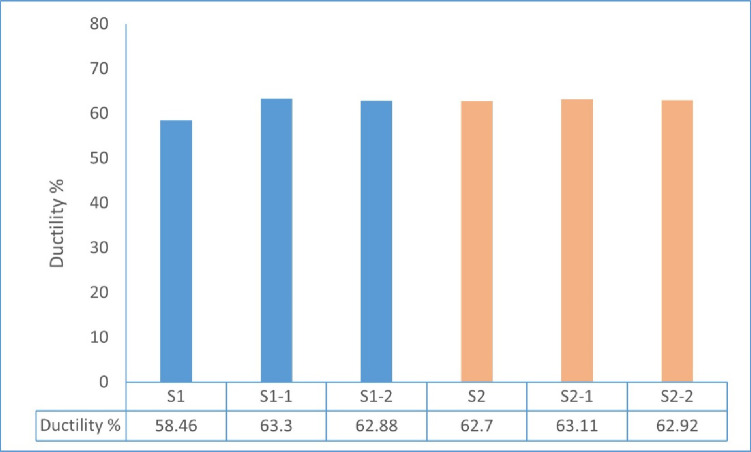
Ductility ratios of tested wall specimens.

The calculated ductility ratios for specimens S1, S1-1, S1-2, S2, S2-1, and S2-2 were 58.46, 63.30, 62.88, 62.70, 63.11, and 62.92, respectively, as illustrated in Fig. 18.

The results show that the GFRP-reinforced walls (S1-1 and S1-2) presented a slightly higher ductility ratio than the steel-reinforced control specimen (S1). Also, the eccentrically loaded samples (S2, S2-1, and S2-2) were found to have similar ductility values as those under concentric loading. In general, the incorporation of GFRP reinforcement maintained a stable ductile response, while load eccentricity did not lead to a noticeable reduction in deformation capacity within the investigated range.

#### Energy absorption

**Fig. 19:**
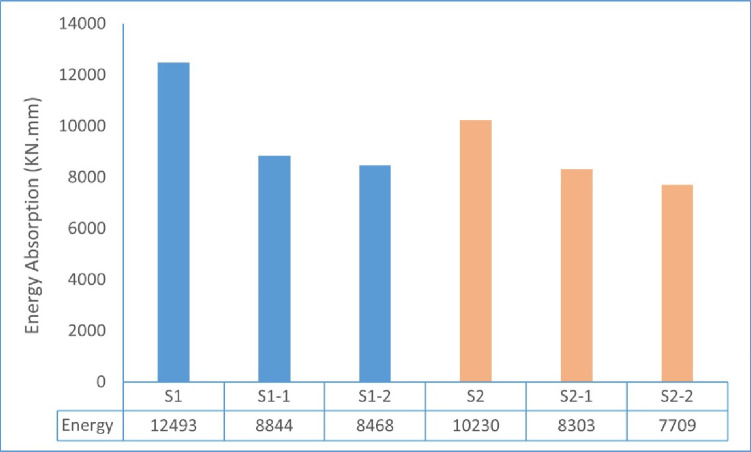
Energy absorption of tested wall specimens.

The area under the load–displacement curve of each tested wall specimen to failure was used to assess the energy absorption. The obtained results are illustrated in Fig. 19. Of tested specimens, the steel-reinforced control wall (S1) showed the maximum energy absorption capacity, which reached 12,493 kN·mm, as evidenced by its ability to sustain higher loads and larger deformations prior to failure. In comparison, the GFRP-reinforced walls (S1-1 and S1-2) demonstrated lower energy absorption values of 8,844 kN·mm and 8,468 kN·mm, respectively.

For the eccentrically loaded specimens, wall S2 recorded an energy absorption capacity of 10,230 kN·mm, while specimens S2-1 and S2-2 exhibited values of 8,303 kN·mm and 7,709 kN·mm, respectively. Overall, the results indicate that both the use of GFRP reinforcement and the presence of load eccentricity influenced the energy absorption capacity of the tested walls. The observed reduction in energy absorption is primarily associated with the lower stiffness and axial load capacity of GFRP-reinforced specimens, as well as the increased deformation demand under eccentric loading.

To enable a consistent comparison between specimens with different reinforcement configurations, the energy absorption results were normalized with respect to the gross volume of the wall specimens. The normalized energy absorption (nE) was calculated as:

$${\mathrm{En}} = {\mathrm{E}}_{exp}/{\mathrm{V}}$$

where $\:{E}_{\mathrm{exp}}$ represents the experimentally measured energy absorption (area under the load–displacement curve up to failure), and $\:V$ is the gross volume of the wall specimen. This normalization facilitates comparative evaluation of the energy absorption capacity by minimizing the influence of specimen size. The normalized results are summarized in Fig. 19 and are discussed in relation to reinforcement type and loading condition.

The lower energy absorption observed in GFRP-reinforced specimens is mainly due to the lower stiffness and limited ductility of GFRP compared to steel. In contrast, steel-reinforced walls can undergo larger deformations before failure, leading to higher energy dissipation. In addition, load eccentricity further reduces energy absorption by increasing bending effects and accelerating crack development.

#### Lateral displacement of tested walls

**Fig. 20:**
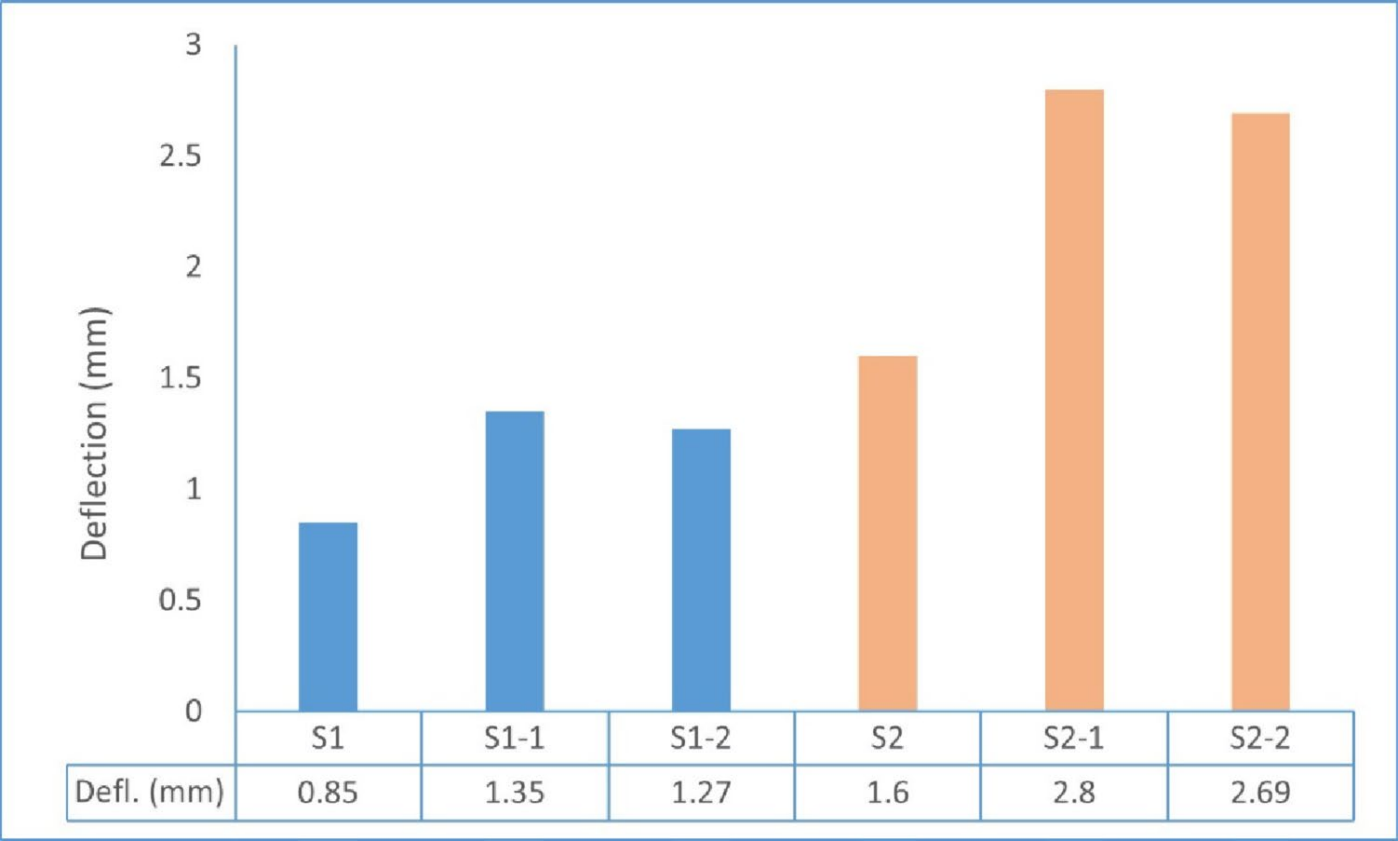
Lateral displacement of tested wall specimens.

The lateral **displacement** of the tested wall specimens was evaluated based on the measured load–deflection responses, as illustrated in Fig. 20. Under concentric loading, the steel-reinforced control specimen S1 presented the least lateral displacement of 0.85 mm, indicating a relatively higher axial stiffness in centered loading conditions. On the other hand, the results of GFRP-reinforced specimens S1-1 and S1-2 indicated their moderate values of deflections of 1.35 mm and 1.27 mm, respectively, as a result of the lower elastic modulus of GFRP bars on the overall deformation response. Eccentrically loaded walls showed noticeably larger displacements than the concentric loading. The deflection of steel-reinforced specimen S2 is 1.60 mm, but for GFRP-reinforced specimens S2-1 and S2-2, the deflection value was 2.80 mm and 2.69 mm respectively. The observations show that load eccentricity is the leading factor for the higher displacement, and GFRP reinforcement increases displacement due to the lower stiffness than steel reinforcement. However, the deflection levels recorded were also within a stable range for all tested specimens.

#### Load–displacement response

**Fig. 21:**
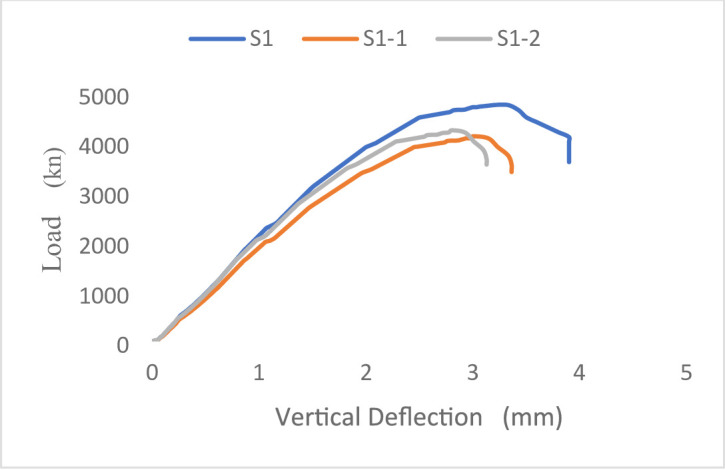
Load-Vertical Deflection curve of Group 1 specimens.

**Fig. 22:**
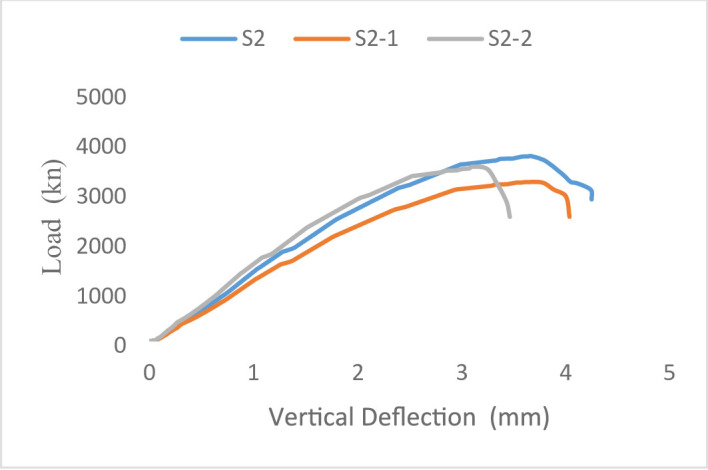
Load-Vertical Deflection curve of Group 2 specimens.

The load–displacement responses of the tested wall specimens are presented in Figs. 21 and 22. The behavior of all specimens remained approximately linear until the first cracking load was applied, resulting in an overall decrease in stiffness as cracking advanced until failure.

The steel-reinforced control specimen S1 for Group G1 (concentric loading) had the highest initial stiffness and ultimate load capacity at 4751 kN when tested. In contrast, the ultimate loads of the GFRP-reinforced specimens S1-1 and S1-2 were 4119 kN and 4237 kN, respectively, and they exhibited a more gradual post-cracking response.

This response was attributed to the lower elastic modulus of GFRP bars, which affected the overall load–displacement response. Moreover, the lack of yielding in GFRP reinforcement restricts stress redistribution within the wall section, resulting in a more gradual reduction in stiffness compared to steel-reinforced specimens. For Group G2 (eccentric loading), all specimens showed increased displacements due to the additional bending moments induced by load eccentricity. The steel-reinforced specimen S2 attained the highest ultimate load of 3748 kN, whereas the GFRP-reinforced specimens S2-1 and S2-2 reached ultimate loads of 3221 kN and 3504 kN, respectively. Despite the reduction in load-carrying capacity, the GFRP-reinforced walls exhibited stable post-cracking behavior without sudden strength loss. Overall, the results indicate that load eccentricity significantly influences the deformation response by increasing displacement demand and reducing axial capacity, while the use of GFRP reinforcement primarily affects stiffness and post-cracking behavior due to its distinct mechanical properties.

**Normalized Load–Displacement Stiffness Comparison**.

**Fig. 23:**
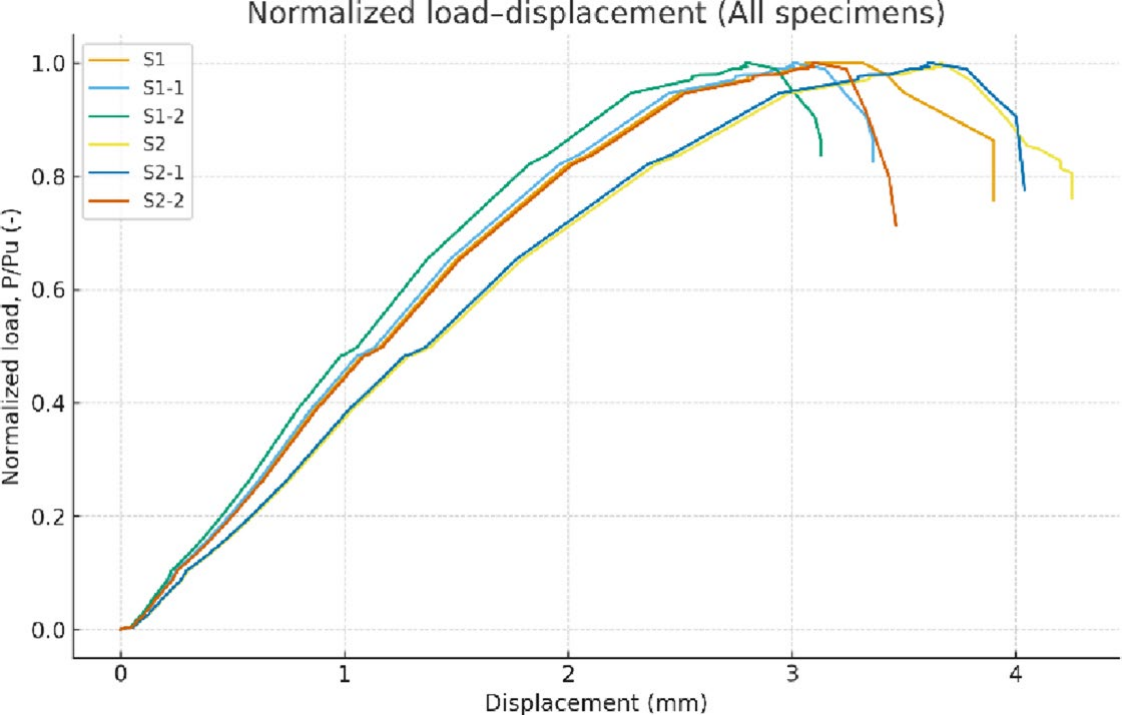
Normalized load–displacement curves (P/Pu vs. displacement) for all tested wall specimens.

Not only the axial load–displacement curves, but also the normalized load–displacement responses were derived by dividing the applied load by the corresponding experimental ultimate load of each specimen (P/Pu). This normalization reduces the influence of differences in ultimate capacity and allows a clearer comparison of the relative deformation trends and post-cracking response among the tested wall specimens on a unified scale, as illustrated in Fig. 23.

**Fig. 24:**
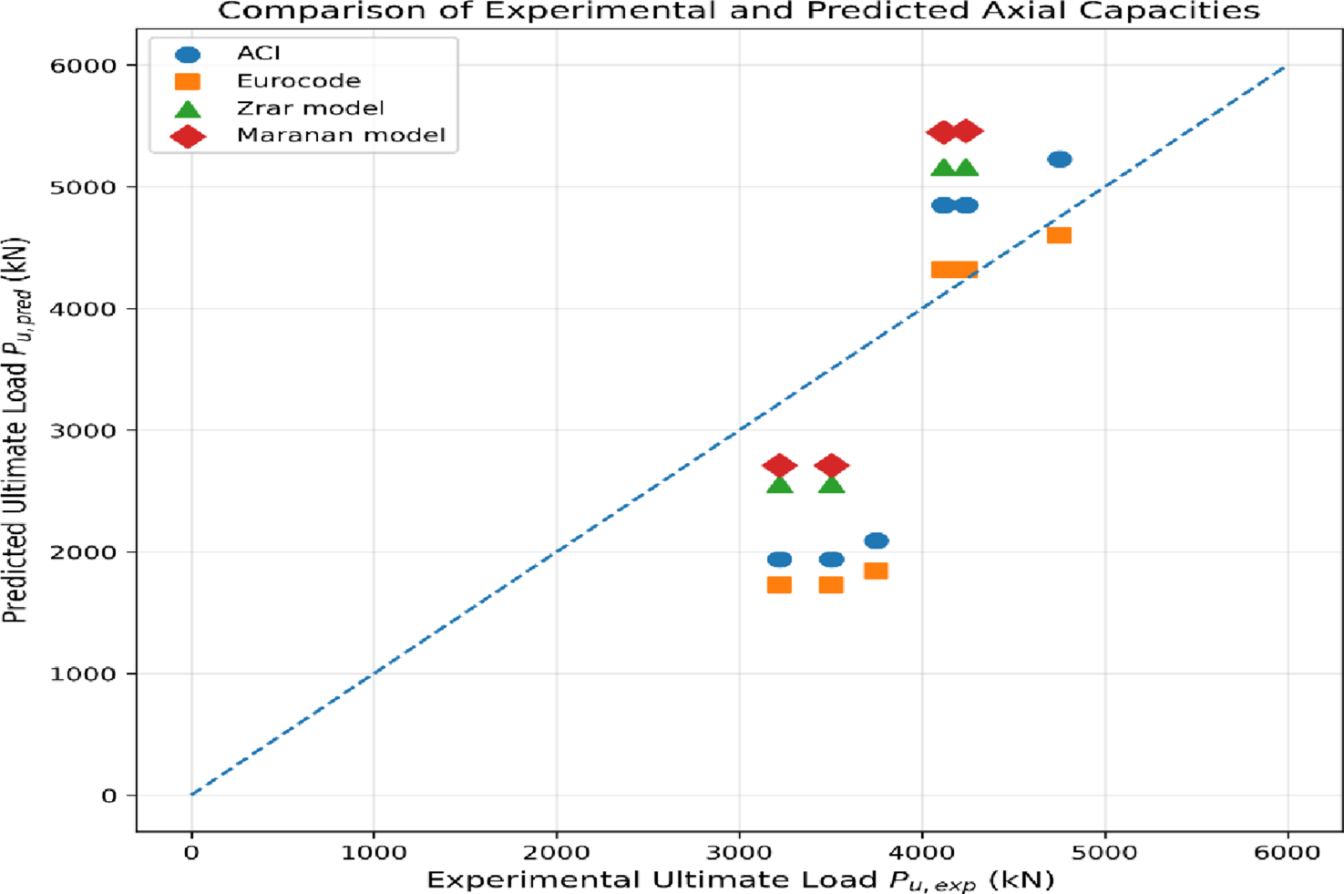
Comparing theoretical and experimental ultimate axial capacities of RC walls using several design methods. The predictions include ACI-based formulations, Eurocode 2 provisions, and analytical models proposed by Zrar and Maranan. Various symbols represent the different design approaches, and the dashed line indicates the ideal condition where predicted and experimental loads are equal.

As illustrated in Fig. 23, all the specimens show similar initial response up to the pre-cracking stage, reflecting about the same initial stiffness. Post-crack behavior, after crack initiation shows the following contrast. Steel-reinforced specimens show higher stiffness and less rapid reducing load performance but while GFRP–reinforced specimens respond steeper as well as exhibit earlier deterioration of stiffness. Furthermore, the effect of load eccentricity is clear because this mechanism decreases the total stiffness and accelerates the departure from linear behavior due to the further bending impact (Fig. 24).

#### Practical implications and field applicability

The results, from a practical perspective, also confirm that GFRP-reinforced concrete walls can act as an efficient and durable substitution for steel-reinforced walls in corrosion-prone environments, for example coastal, marine and industrial sites. Although GFRP bars are usually 20–30% more expensive per unit length than steel, the overall life-cycle cost is lower because they do not require significant corrosion maintenance and extend their service life. In applications for the field, GFRP walls are considered to be especially helpful when lightweight reinforcements, non-magnetic properties or electrical insulation, may be desired (e.g., hospitals, substations and coastal retaining walls). The relatively small decrease in load tolerance (≈ 6.5–14.1%) presented in this work can be easily compensated with only small revisions in section and conservative design to ensure satisfactory serviceability due to the better ductility and crack management. Therefore, the integration of GFRP reinforcement in RC walls offers a technically feasible and economically sustainable solution for long-term durability in aggressive environments.

This paper demonstrated a clear and definitive reduction in axial capacity that occurred with the implementation of GFRP bars replacing steel reinforcement in RC walls. This could help design engineers to manage such reductions during the design stage, through the use of proper safety margins, or minor adjustment of wall dimensions. Similarly, the comparison with ACI 440.1R, CSA S806, and Eurocode provisions along with the proposed modification factor helps provide a more realistic estimation of the axial capacity of GFRP-reinforced walls. Rationally speaking, the mixed experimental and numerical approach used provides a robust framework for further research. The established finite element model can effectively study the essential parameters, reinforcement ratio, wall geometry, load eccentricity and so on, without the need for long experimental schedule. Furthermore, while the concrete strain recorded was marginally greater than the widely adopted ultimate value (≈ 0.0035) owing to higher-grade concrete and isolated strain concentrations, failure was distinctly monitored by concrete crushing, as verified from empirical evidence. With the exception of the current reports, this study adds to the relatively small number of studies investigating GFRP reinforced walls under axial loading and allows for further development of analytical methods and improvement of design provisions. The outcome of this work could have immediate practical application on designing, and assessing, for example, the design and evaluation of these structural products and suggest how the choice of type of reinforcement influences the performance of the buildings.

#### Benchmarking and evaluation of code-based and analytical models for RC walls under concentric and eccentric axial loading

Table 8 summarizes the ultimate load capacities of steel- and GFRP-reinforced reinforced concrete (RC) walls, together with their corresponding geometric and mechanical properties. The table includes section dimensions, eccentricity values, reinforcement areas, and elastic moduli for both steel and GFRP reinforcement systems. In addition, the experimentally measured ultimate loads (**P**_exp_) are compared with the predicted capacities obtained from the **Zrar** and **Meranan** analytical models. The ratios between experimental and predicted loads are also reported to assess the accuracy and reliability of these models in capturing the structural response of RC walls reinforced with GFRP in comparison with conventionally steel-reinforced walls, as well as the influence of load eccentricity and reinforcement type on the ultimate load capacity.

**Methodology for analytical axial capacity calculations**.

The analytical axial capacities reported in Table 8 were calculated using analytical models proposed by Zrar et al. and Maranan et al. for reinforced concrete members reinforced with FRP bars and subjected to concentric and eccentric axial loading. The calculations were based on force equilibrium and strain compatibility principles, while explicitly accounting for the effect of load eccentricity.

According to the Zrar model, the ultimate axial capacity is primarily attributed to the concrete contribution and is reduced as a function of load eccentricity, as expressed by:

$$\:{P}_{Zrar}=\beta\:{f}_{c}^{{\prime\:}}{A}_{c}\left(1-\frac{e}{t}\right)$$

where $\:{f}_{c}^{{\prime\:}}$ is the concrete compressive strength, $\:{A}_{c}$ is the effective concrete area, $\:e$ is the load eccentricity, $\:t$ is the wall thickness, and $\:\beta\:$ is an experimentally calibrated coefficient accounting for material variability.

The Maranan model evaluates the axial capacity using an effective concrete compressive strength that accounts for eccentric loading effects, such that:

$$\:{P}_{Maranan}={f}_{c,eff}^{{\prime\:}}{A}_{c}$$

where the effective compressive strength $\:{f}_{c,eff}^{{\prime\:}}$ is expressed as a function of the eccentricity-to-thickness ratio $\:\left(e/t\right)$. All analytical predictions were computed using the experimentally measured material properties and geometric dimensions of the tested RC wall specimens.

**Table 8:** Ultimate load capacity for steel- and GFRP-reinforced RC walls.

Column Specimen	ƒς	B (mm)	T (mm)	e (mm)	As (mm²)	Es (Mpa)	Af (mm²)	Ef (Mpa)	*P* exp (KN)	*P* zrar (KN)	*P* meranan (KN)	*P* EX /*P* Zrar	*P* EX /*P* Meranan
**S1**	50	800	150	0	1131	200,000	-	-	4751	-	-	-	-
**S1-1**				0	-	-	1131	45,000	4119	5166	5451	0.797	0.76
**S1-2**				0	-	-	1131	45,000	4237	5166	5461	0.820	0.78
**S2**				37.5	1131	200,000	-	-	3748	-	-	-	-
**S2-1**				37.5	-	-	1131	45,000	3221	2560	2711	1.26	1.19
**S2-2**				37.5	-	-	1131	45,000	3504	2560	2711	1.37	1.29

The parameters listed in Table 8 are defined as follows: fcf_cfc​ denotes the concrete compressive strength; BBB and TTT represent the wall length and thickness, respectively; and eee is the load eccentricity. The terms AsA_sAs​ and EsE_sEs​ correspond to the area and elastic modulus of steel reinforcement, while AfA_fAf​ and EfE_fEf​ refer to those of GFRP reinforcement.

The experimental ultimate load is denoted by PexpP_{exp}Pexp​, whereas PZrarP_{Zrar}PZrar​ and PMerananP_{Meranan}PMeranan​ represent the predicted ultimate loads based on the analytical models proposed by Zrar and Meranan, respectively. The ratios Pexp/PZrarP_{exp}/P_{Zrar}Pexp​/PZrar​ and Pexp/PMerananP_{exp}/P_{Meranan}Pexp​/PMeranan​ are used to evaluate the agreement between the experimental results and the corresponding model predictions.

**Comparison between experimental results and code-based predictions**.

The axial load-carrying capacities obtained from the experimental program were compared with the corresponding predictions calculated using the American and European design codes. The analytical values were computed according to the ACI-based formulations and Eurocode 2 provisions, employing the experimentally measured material properties and geometric characteristics of the tested wall specimens.

**Methodology for code-based axial capacity calculations**.

The code-based axial capacities presented in Table 9 were calculated in accordance with the provisions of ACI 318 in conjunction with ACI 440.1R and CSA S806 and Eurocode 2. The calculations were performed using the experimentally measured material properties and geometric characteristics of the tested wall specimens.

According to the ACI 440.1R and CSA S806 formulation, the nominal axial capacity of concentrically loaded RC walls reinforced with steel bars is calculated as:

$$\:{P}_{n}=0.85{f}_{c}^{{\prime\:}}({A}_{g}-{A}_{s})+{f}_{y}{A}_{s}$$

whereas for GFRP-reinforced members, the contribution of GFRP bars in compression is neglected in accordance with ACI 440.1R and CSA S806 recommendations, and the axial capacity is expressed as:

$$\:{P}_{n}=0.85{f}_{c}^{{\prime\:}}{\:A}_{g}$$

Eurocode 2 evaluates the axial capacity based on the design concrete strength and reinforcement contribution, as expressed by:

$$\:{P}_{n}={\alpha\:}_{cc}{f}_{cd}{A}_{c}+{f}_{yd}{A}_{s}$$

For eccentrically loaded walls, the reduction in axial capacity prescribed by each code was applied, and the experimental-to-predicted capacity ratios were subsequently used to assess the accuracy and conservatism of each design approach.

**Direct comparison with design codes**.

Table 9 presents a detailed comparison between the experimentally measured ultimate axial loads and the corresponding predictions obtained from the ACI 440.1R and CSA S806 and Eurocode formulations for both concentric and eccentric loading conditions. The ratios between experimental and predicted capacities are also reported to quantify the accuracy and conservatism of each design approach.

**Table 9:** Comparison between experimental and code-predicted axial capacities of RC walls.

Specimen	Loading type	Pexp (kN)	PACI (kN)	PEC (kN)	Pexp/PACI	Pexp/PEC
S1	Concentric	4751	5229.87	4605	0.91	1.03
S1-1		4119	4849.86	4320	0.85	0.95
S1-2		4237	4849.86	4320	0.87	0.98
S2	Eccentric	3748	2091.95	1842	1.79	2.03
S2-1		3221	1939.94	1728	1.66	1.86
S2-2		3504	1939.94	1728	1.81	2.03

P_exp: Experimental ultimate load.

P_ACI: Predicted axial load according to ACI provisions.

P_EC: Predicted axial load according to Eurocode.

As demonstrated by Table 9, ACI-based predictions for concentrically loaded walls generally overestimate experimental axial capacities; Pexp/PACI ratios are consistently lower than unity. In contrast, Eurocode predictions are in good agreement with experimental results under concentric loading. For eccentrically loaded specimens, both ACI 440.1R and CSA S806 as well as Eurocode formulations yield relatively conservative predictions.

The results obtained in this study are broadly in line with those observed in previous works on FRP-reinforced concrete members. Similar trends have been reported, showing that the axial capacity for GFRP-reinforced elements is relatively lower than steel-reinforced ones, mainly owing to the lower elastic modulus of the GFRP.

Moreover, the observed reduction in load capacity under eccentric loading conditions is consistent with the established behavior of compression members subjected to combined axial load and bending. Despite this agreement, the present study offers additional insight into the response of GFRP-reinforced RC wall systems, which have received comparatively less attention in the literature than beams and columns.

**Statistical assessment and proposed design modification**.

To identify systematic trends and support the development of practical design recommendations, a statistical evaluation of the experimental-to-predicted capacity ratios was performed. The mean values, scatter, and coefficients of variation are summarized in Table  10.

**Table 10:** Statistical summary of experimental-to-predicted axial capacity ratios.

Loading condition	Design code	Mean (μ)	Min	Max	Std. Dev. (σ)	COV (%)
Concentric	ACI	0.88	0.85	0.91	0.03	3.4
Concentric	Eurocode	0.99	0.95	1.03	0.04	4.0
Eccentric	ACI	1.75	1.66	1.81	0.08	4.6
Eccentric	Eurocode	1.97	1.86	2.03	0.09	4.5

**Statistical evaluation procedure**.

To evaluate the accuracy and reliability of the analytical models and code-based predictions, the experimental-to-predicted axial capacity ratio was calculated for each specimen as:

$$\:\mathrm{EXP/Pred}=\frac{{P}_{exp}}{{P}_{pred}}$$

where $\:{P}_{exp}$ is the experimentally measured ultimate axial load and $\:{P}_{pred}$ is the corresponding predicted value obtained from analytical or code-based formulations.

The statistical parameters reported in Table 10 were determined using the mean value and standard deviation of the experimental-to-predicted ratios. The standard deviation was calculated as:

$$\:\sigma\:=\sqrt{\frac{1}{n-1}{\sum\:}_{i=1}^{n}({x}_{i}-\mu\:{)}^{2}}$$

where $\:{x}_{i}$ is the experimental-to-predicted ratio for each specimen, $\:\mu\:$ is the mean value of the ratios, and $\:n$ is the total number of specimens. These statistical indicators were used to quantify the dispersion and consistency of each predicti.

The statistical results confirm that the overestimation observed in the ACI 440.1R predictions for concentrically loaded GFRP-reinforced RC walls is systematic rather than random. Accordingly, a modification factor of 0.88 is proposed to improve the accuracy of the ACI-based axial capacity prediction.

The modified ACI 440.1R axial capacity expression for concentrically loaded RC walls reinforced with GFRP bars can be written as:

$$\:{P}_{n,\mathrm{mod}}=0.88\times\:{P}_{n,\mathrm{ACI}}$$

where $\:{P}_{n,\mathrm{ACI}}$is the nominal axial capacity predicted using the original ACI 440.1R formulation. The proposed modification considerably minimizes the prediction bias alongside an acceptable level of conservatism, making the ACI 440.1R approach more suitable for GFRP-reinforced RC wall systems.

**Engineering implications**.

These direct and statistical comparisons indicate the need for minor calibration of ACI-based formulations used in concentrically loaded GFRP-reinforced RC walls. The proposed modification factor offers a simple and practical correction that improves prediction accuracy without compromising safety.

## Nonlinear finite element analysis

The nonlinear finite element analysis (NLFEA) techniques were applied with ABAQUS/CAE 2020 to simulate the behavior of reinforced concrete (RC) walls reinforced with steel and GFRP bars subjected to concentric and eccentric axial loading. The FE models developed were designed to represent the load–displacement response, cracking pattern, and ultimate load capacity from the experiment^46^.

### Constitutive stress–strain relationships

**Fig. 25:**
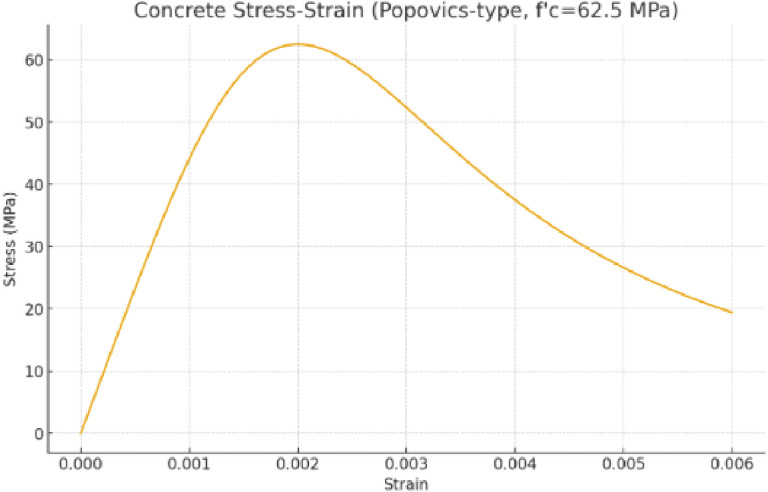
Stress–strain relationship of concrete according to the Popovics model.

Material constitutive behavior for concrete and reinforcement was defined using idealized uniaxial stress–strain relationships in the experimental interpretation and FE modelling. The compressive stress–strain curve of concrete was obtained as mean 28-day cube strength (62.5 MPa) and transformed into the equivalent uniaxial model with nonlinear ascending branch to peak stress and softening (Fig. 25). The used relationship is taken from the Popovics model, which has been validated to capture nonlinear compressive response of concrete accurately.

Steel reinforcement was idealized according to a bilinear elastic–plastic model with Eₛ = 200 GPa and f_γ_ = 420 MPa, while GFRP bars were specified as linear elastic up to rupture with E_f = 50 GPa and f_fu = 1200 MPa. Those constitutive laws remained consistent when interpreting measurements of the axial strain data obtained by the strain gauges in the laboratory and when determining the input material parameters for the concrete damaged plasticity (CDP) model, in ABAQUS.

**Damage Parameters in CDP Model**.

The compressive and tensile damage parameters ($\:{d}_{c}$ and $\:{d}_{t}$) in the CDP model were computed from the descending branches of the uniaxial stress–strain curves. Each damage value was defined as the loss of effective stiffness relative to the undamaged elastic response according to:

$$\:d=1-\frac{\sigma\:}{E\epsilon\:}$$

where $\:\sigma\:$and $\:\epsilon\:$ represent the stress and strain at the same point on the post-peak curve. The resulting tabulated $\:(\epsilon\:,\sigma\:,d)$ values for compression and tension were directly input into ABAQUS to reproduce stiffness degradation associated with concrete crushing and cracking.

### Reinforced concrete wall modelling

**Fig. 26:**
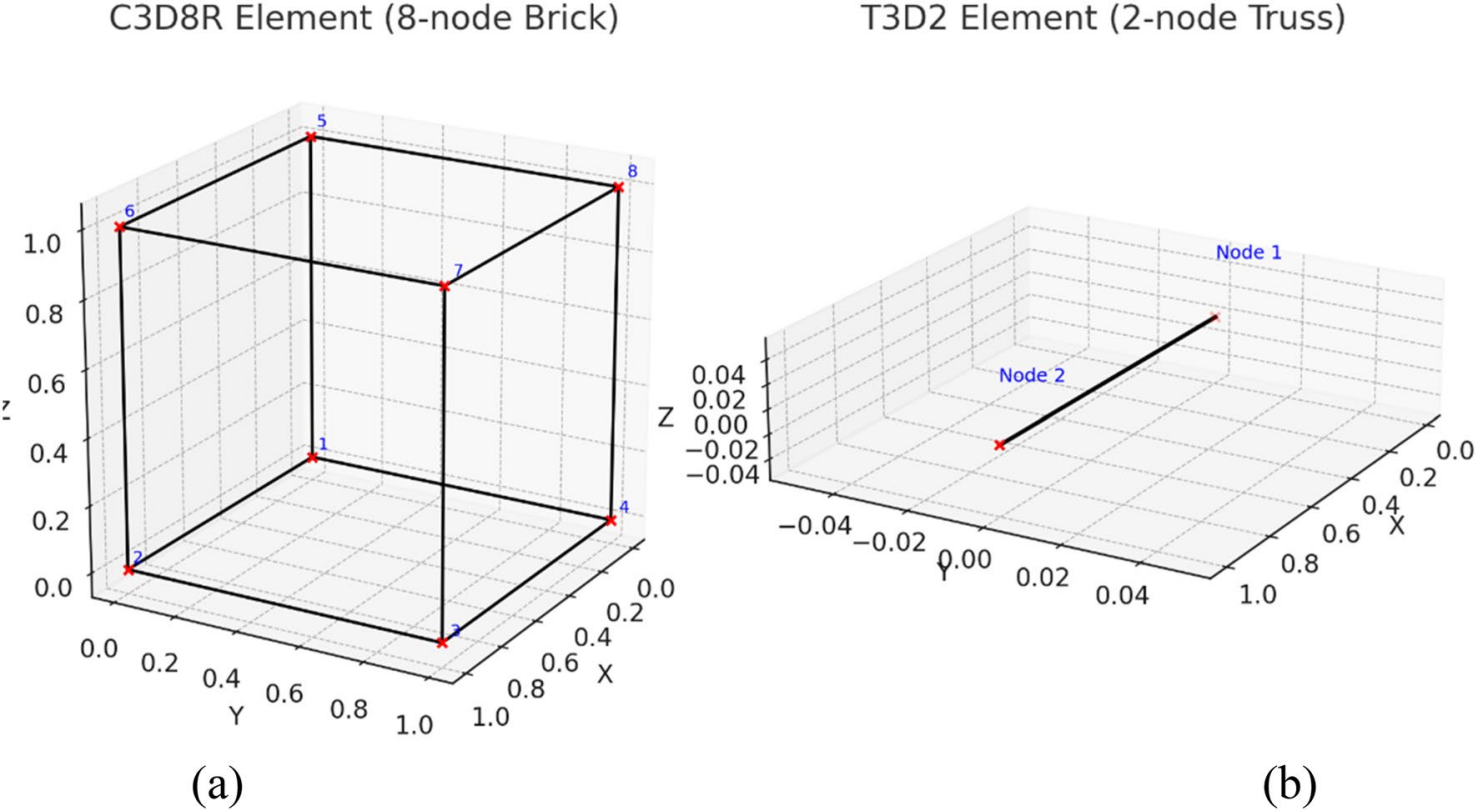
Finite element types used in ABAQUS: (**a**) C3D8R 8-node brick element for concrete, (**b**) T3D2 2-node truss element for reinforcement.4.3 Verification of model.

- Concrete: modeled using 8-node reduced-integration brick elements (C3D8R), as shown in Fig. 26a.
- Nonlinear concrete response: defined by the Concrete Damaged Plasticity (CDP) model.
- Reinforcement: modeled using T3D2 truss elements (steel or GFRP) fully embedded into the concrete matrix using the embedded region constraint, as shown in Fig. 26b, the embedded element approach assumes a perfect bond between the reinforcement and the surrounding concrete. Although this simplification neglects local bond–slip behavior at the GFRP–concrete interface, it is widely used in the analysis of compression-dominated RC elements. The validity of this assumption is supported by the close agreement between the experimental and numerical results in terms of ultimate load capacity, load–displacement response, and crack patterns.
- Mesh: structured with an element size of 50 mm, which provided good accuracy and computational efficiency.
- Loading and support plates: represented by rigid surfaces (R3D4) coupled to reference points (RPs)^45^.

- The FE model was validated against experimental curves in terms of first-crack load, ultimate load, and load–displacement response.
- Analysis used displacement control via Static General step with NLGEOM = ON.
- Newton–Raphson iteration was applied until convergence; loss of convergence corresponded to failure.
- In the nonlinear finite element analysis, failure was identified based on the loss of convergence of the Newton–Raphson iterations, which coincided with the localization of compressive and tensile strains in the critical regions of the wall. This stage corresponds to the formation of continuous cracking zones and crushing in the concrete compression area, marking the ultimate load condition.
- The error margin between experimental and numerical predictions remained less than 7%, confirming the reliability and accuracy of the adopted FE model.
- The FE predictions showed strong agreement with experimental results, with deviations < 7% in ultimate load and stiffness.

### Material properties

**Concrete**.

- Elastic modulus: Ec = 4400√fcu = 34,100 MPa (for fcu = 60 MPa).
- Poisson’s ratio: ν = 0.20.
- CDP parameters: dilation angle = 35°, eccentricity = 0.1, fbo/fco = 1.16, K = 0.667, viscosity = 0.0005.

**Steel reinforcement**.

- Elastic modulus: Es = 200 GPa.
- Yield stress: fy = 420 MPa.
- Poisson’s ratio: ν = 0.30.
- Modeled as elastic–plastic with isotropic hardening.

**GFRP reinforcement**:

- Elastic modulus: Ef = 50 GPa.
- Tensile strength: fu = 1200 MPa.
- Poisson’s ratio: ν = 0.25.
- Modeled as linear elastic up to rupture (brittle behavior).

### Boundary conditions and loading

- Supports: the base plate RP was fully restrained .
- Loading: applied to the top plate RP through vertical deflection control.
- Concentric loading: RP located at the centroid of the wall cross-section.
- Eccentric loading: RP was offset from the centroid by the experimental eccentricity.
- Contact: defined as hard contact in the normal direction with penalty friction (µ = 0.3).

### Mesh sensitivity analysis

**Fig. 27:**
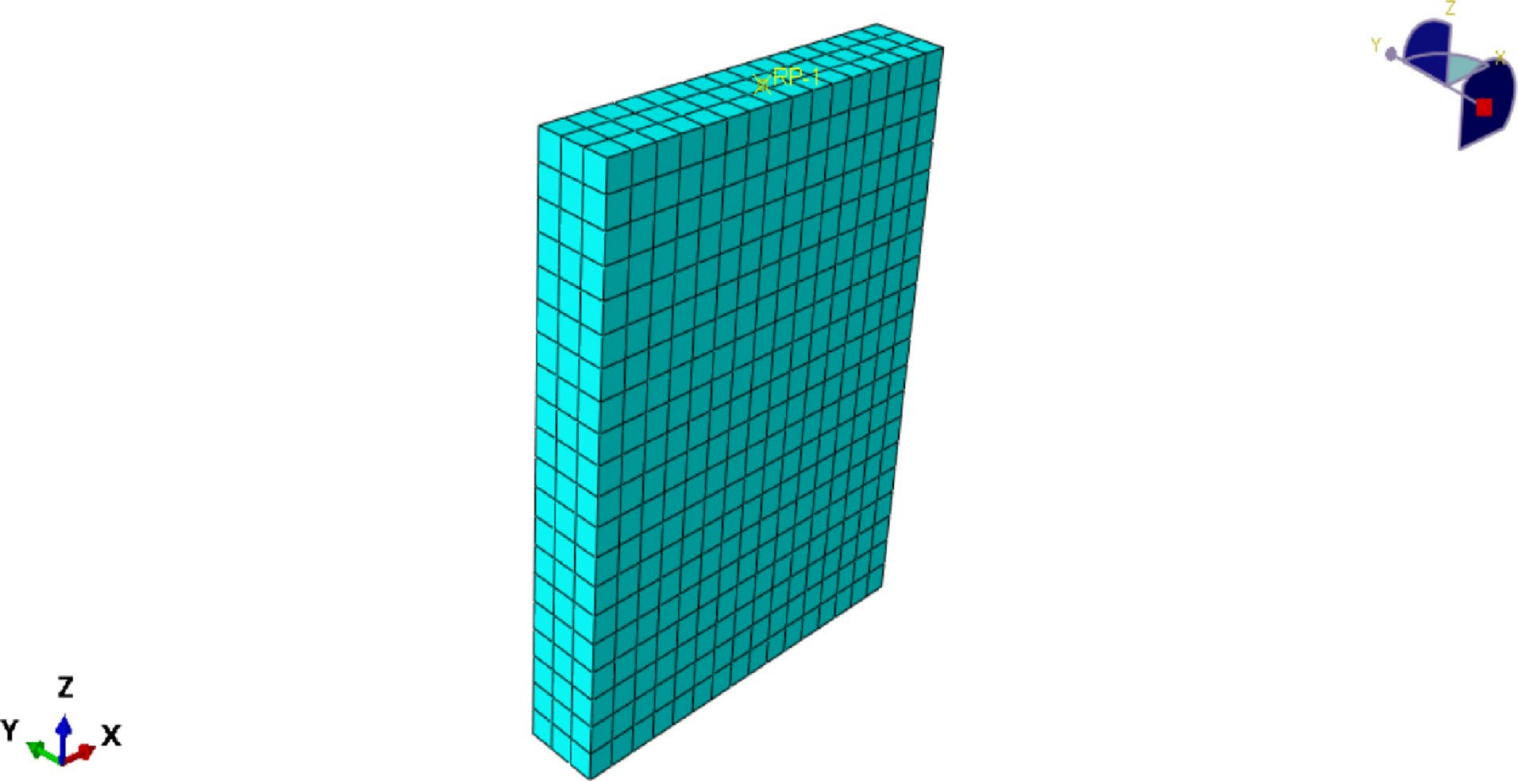
Meshed finite element model of RC wall using C3D8R solid elements in ABAQUS.

**Fig. 28:**
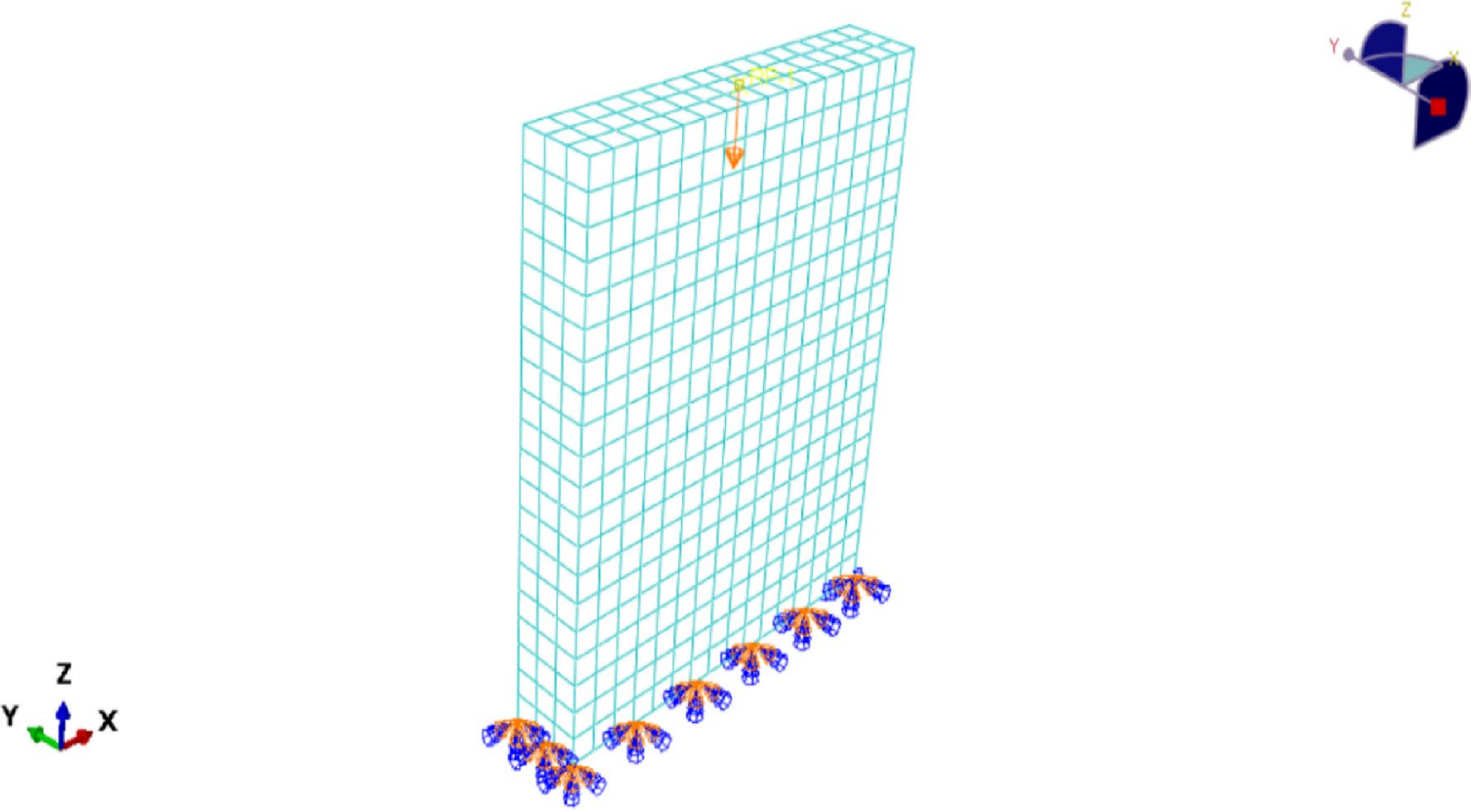
Finite element model of RC wall in ABAQUS showing meshing, boundary conditions, and applied axial load.

- Mesh sizes of 30, 50, and 75 mm were examined.
- Ultimate load variations were within 3–5%, and displacement predictions varied by < 6%.
- A mesh size of 50 mm was selected as the optimal balance between computational cost and accuracy.
- A mesh convergence study with element sizes of 30, 50, and 75 mm was performed in order to investigate mesh refinement on the numerical predictions. The difference between the ultimate load values of each mesh was less than 6%, indicating that the chosen mesh produced stable and valid results. The influence of mesh size on anticipated ultimate load is summarized in Table 11 and demonstrated with the numerical response, and the mesh size is determined to converge around the 50 mm mesh, which is taken as the optimum for our analysis (Figs. 27, 28).

**Table 11:** Effect of mesh size on predicted ultimate load.

Mesh size (mm)	Ultimate load (kN)	Variation (%)
**30**	4820	-
**50**	4780	0.8
**75**	4660	0.3

### NLFEA ultimate failure load

Ultimate loads obtained from the NLFEA were determined for the two groups of wall specimens subjected to concentric and eccentric loading, as summarized in Table 12. For the first group (G1, concentric loading), the control specimen S1 recorded a failure load of 4852.15 kN. The hybrid GFRP-reinforced specimens S1-1 and S1-2 exhibited slightly lower ultimate loads of 4609.7 kN and 4661.5 kN, respectively, with reductions in the range of 3–5% compared with the control. This indicates that the replacement of steel reinforcement with GFRP bars in concentric walls slightly affected the ultimate capacity, while maintaining comparable structural performance.

For the second group (G2, eccentric loading), the control specimen S2 exhibited a failure load of 3541.42 kN. In contrast, the GFRP-reinforced specimens S2-1 and S2-2 recorded ultimate loads of 3228.63 kN and 3392.35 kN, respectively, corresponding to reductions of 8.8% and 4.2% relative to the control. Despite the reduced capacity, the eccentric walls reinforced with GFRP showed adequate load-carrying performance and stable post-cracking behavior.

A direct comparison between experimental and numerical results revealed excellent agreement, with the numerical predictions deviating from the experimental ultimate loads by an average of only ± 7%. This confirms the capability of the ABAQUS model to capture the nonlinear response and failure mechanisms of the tested walls with high accuracy (Table 13).

**Table 12:** NLFEA results of first crack load, failure load, and deflection for tested wall specimens.

Group	Specimen	First crack load (KN)	Failure load (KN)	Deflection at Pu (mm)
G1 (Concentric)	S1	2615.33	4852.15	2.4
	S1-1	2495.45	4609.7	2.45
	S1-2	2496.98	4661.5	2.55
G2 (Eccentric)	S2	1663.83	3541.42	2.2
	S2-1	1466.04	3228.63	2
	S2-2	1639.01	3392.35	2.1

**Table 13:** Experimental and FE crack pattern comparison.

Specimen	Experimental observation	FE crack contour analysis
S1	Mid-height vertical cracks, crushing near top edge.	Similar crack band in tension area, localized crushing at top
S1-1	Wider cracks near center, minor diagonal cracks	Comparable pattern with slightly delayed crack initiation
S1-2	Diagonal cracks due to eccentric compression	FE contours show same diagonal crack path with reduced tensile damage
S2	FE contours show same diagonal crack path with reduced tensile damage	Matching crack angles and distribution in tension zone
S2-1	Matching crack angles and distribution in tension zone	Combined vertical and inclined cracks
S2-2	FE contours captured identical diagonal crack zone and gradual propagation	Fine diagonal cracks concentrated near compression edge

### NLFEA load–displacement response

**Fig. 29:**
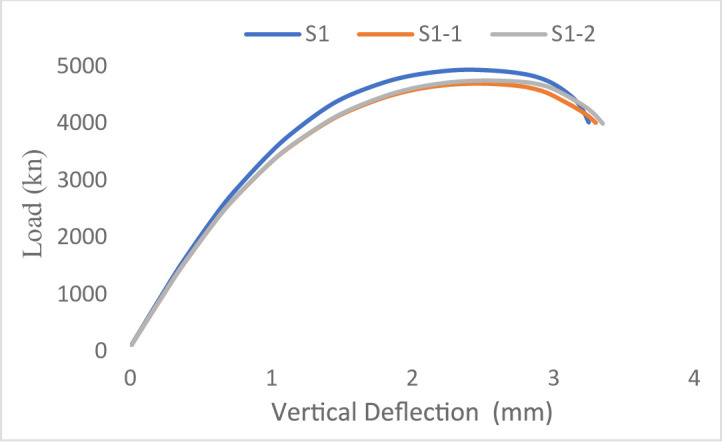
NLFEA load–Vertical Deflection response of Group 1.

**Fig. 30:**
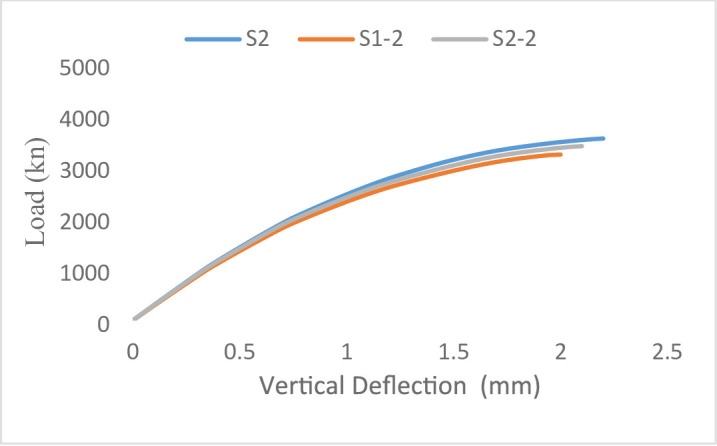
NLFEA load–Vertical Deflection response of Group 2.

ABAQUS nonlinear finite element analysis (NLFEA): load–displacement curves were also obtained for the concentric (G1) and eccentric (G2) wall type. The findings were in good agreement with the experimental data and the curves recorded the initial stiffness, peak load, and post-peak softening. The control specimen (S1) of Group 1 obtained the highest load capacity, while the other two, GFRP reinforced specimens (S1-1 and S1-2) displayed slightly decreased strength and with similar displacement at failure. The eccentric loading at Group 2 was reported to result in stiffness and ultimate strength being lower after eccentric loading than G1 which are also in agreement to those observed in the tests. In conclusion, the NLFEA results clearly confirmed the ability of ABAQUS to predict the nonlinear behavior of RC walls with GFRP reinforcement in the simulation realistically. The NLFEA loads and displacements of the specimens of the tested groups 1 and 2 were displayed in Figs. 29 and 30 respectively.

### Crack pattern and failure mode (NLFEA)

Cracks in both S1 and S2 control specimens originated in the tension zones and propagated progressively with load increase, represented in Fig. 28. NLFEA results showed that cracks were distributed mostly at the lower region under concentric loading, while eccentric loading allowed for the formation of localized cracks close to the compression edge. Specimens equipped with GFRP bars had a finer and generally more uniform distribution of cracks than steel-reinforced specimens. Tensile failure of the reinforcement combined with progressive concrete cracking was the predominant failure mode in all cases, confirming the results obtained during the experiment. In addition, the numerical predictions were in strong agreement with the experimental cracking patterns, thus verifying the reliability of these developed FE models. This was done through a joint comparison of the results between the experimental crack and numerical crack of both the groups. The finite element (FE) crack contours replicated the main experimental cracks, which included vertical tension cracks at mid-height and diagonal compression cracks located close to the loaded corners.

The comparison confirmed that the CDP model in ABAQUS effectively simulated observed cracking initiation, propagation, and failure zones in the laboratory tests. The concordance of experimental and numerical crack trends demonstrates the soundness of the FE model in obtaining the true cracking properties of steel- and GFRP-reinforced walls.

**SDEG Crack Contours and Color Legend.**

**Fig. 31:**
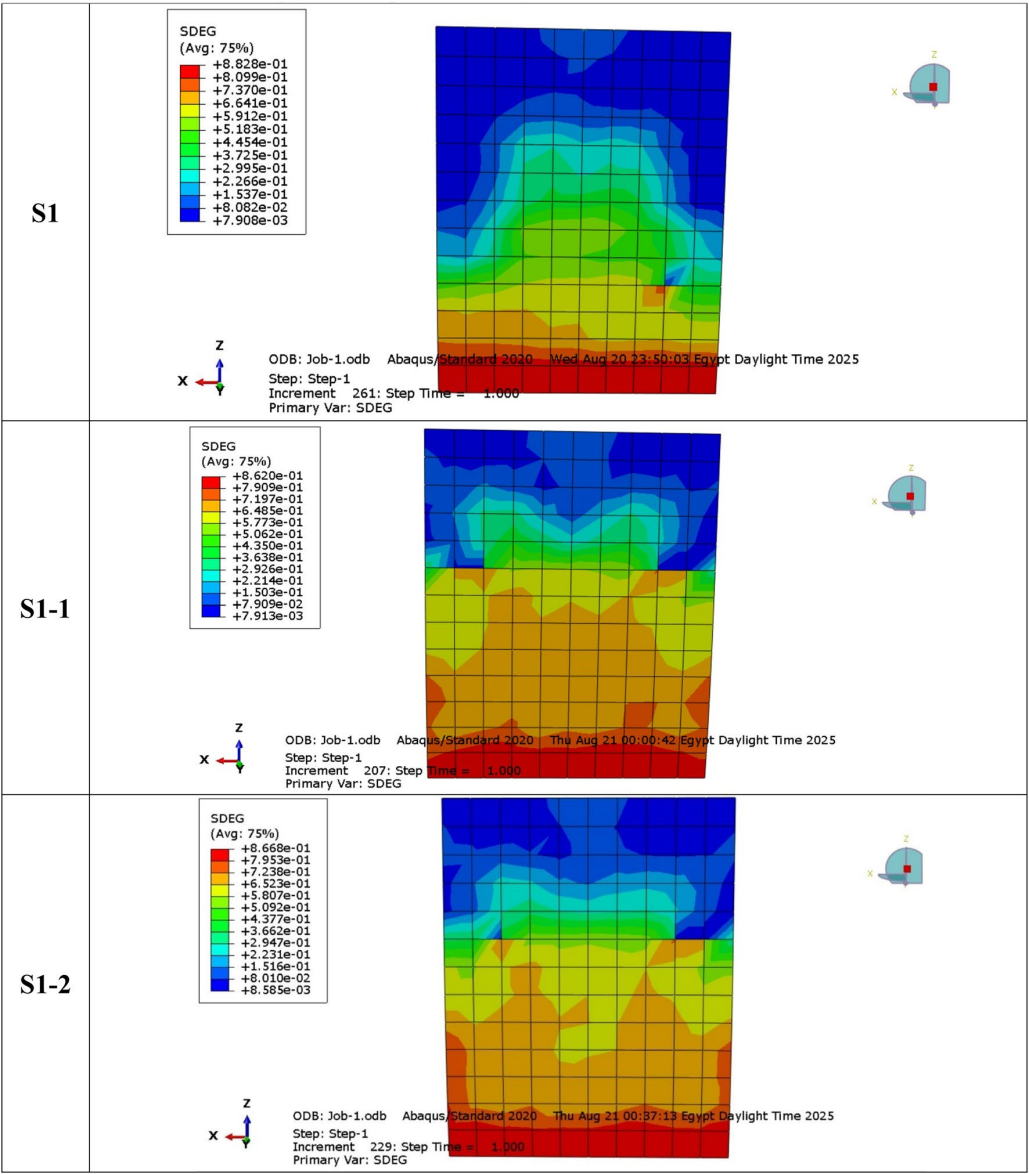

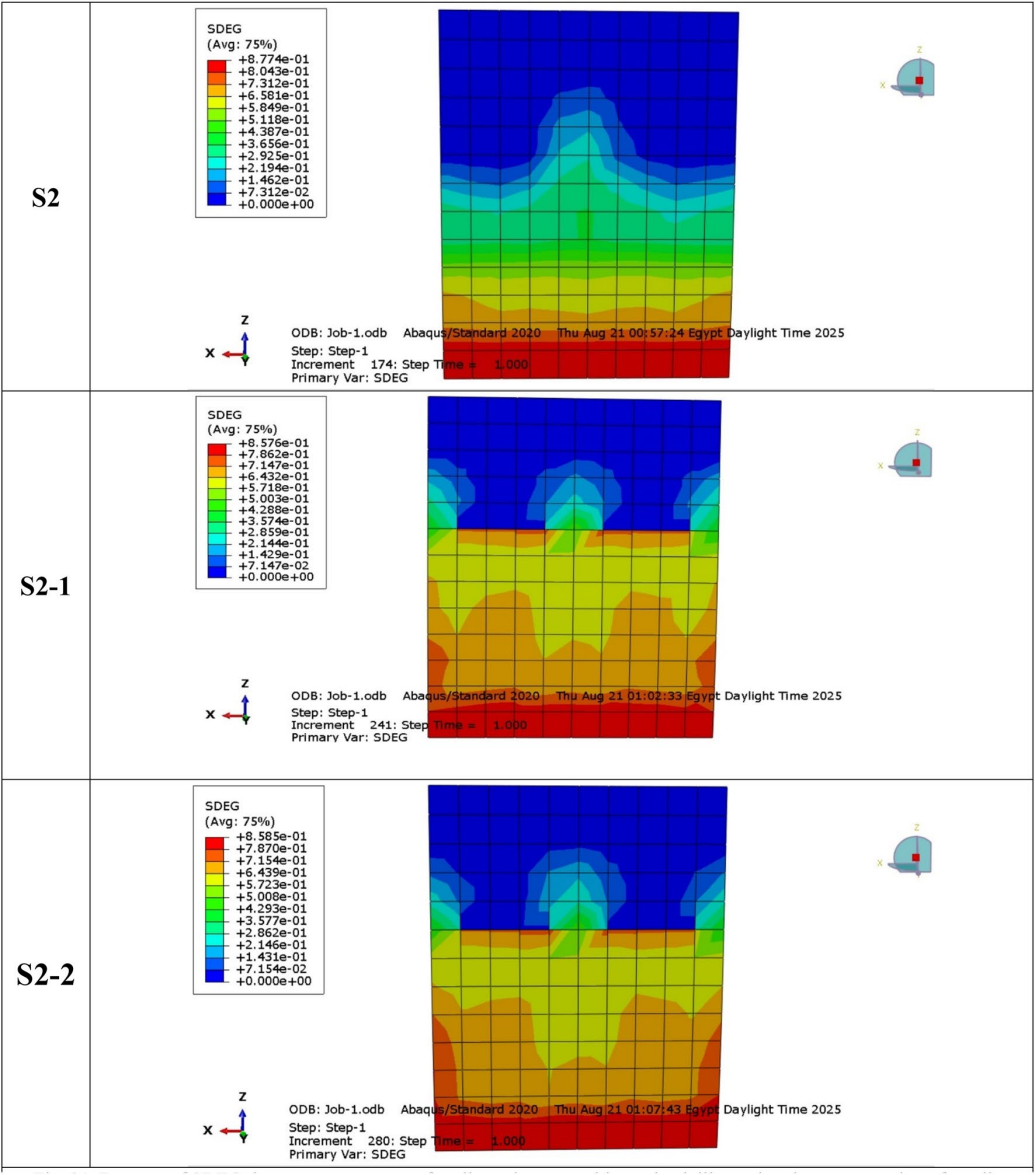
Damage of SDEG damage contour maps for all specimens at ultimate load, illustrating the concentration of tensile damage near the compression toe and its spread toward mid height.

**Fig. 32:**
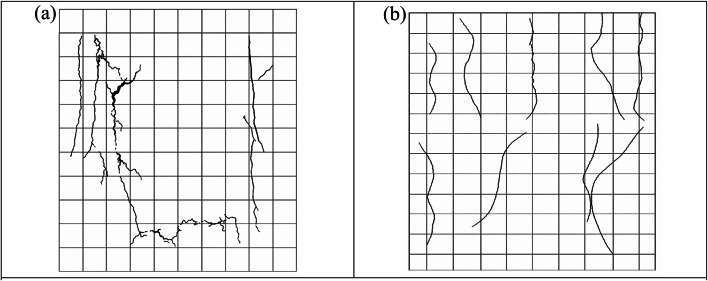
Sample of NLFEA results of cracks: (**a**) walls under concentric loads; (**b**) walls under eccentric loads.

**Fig. 33:**
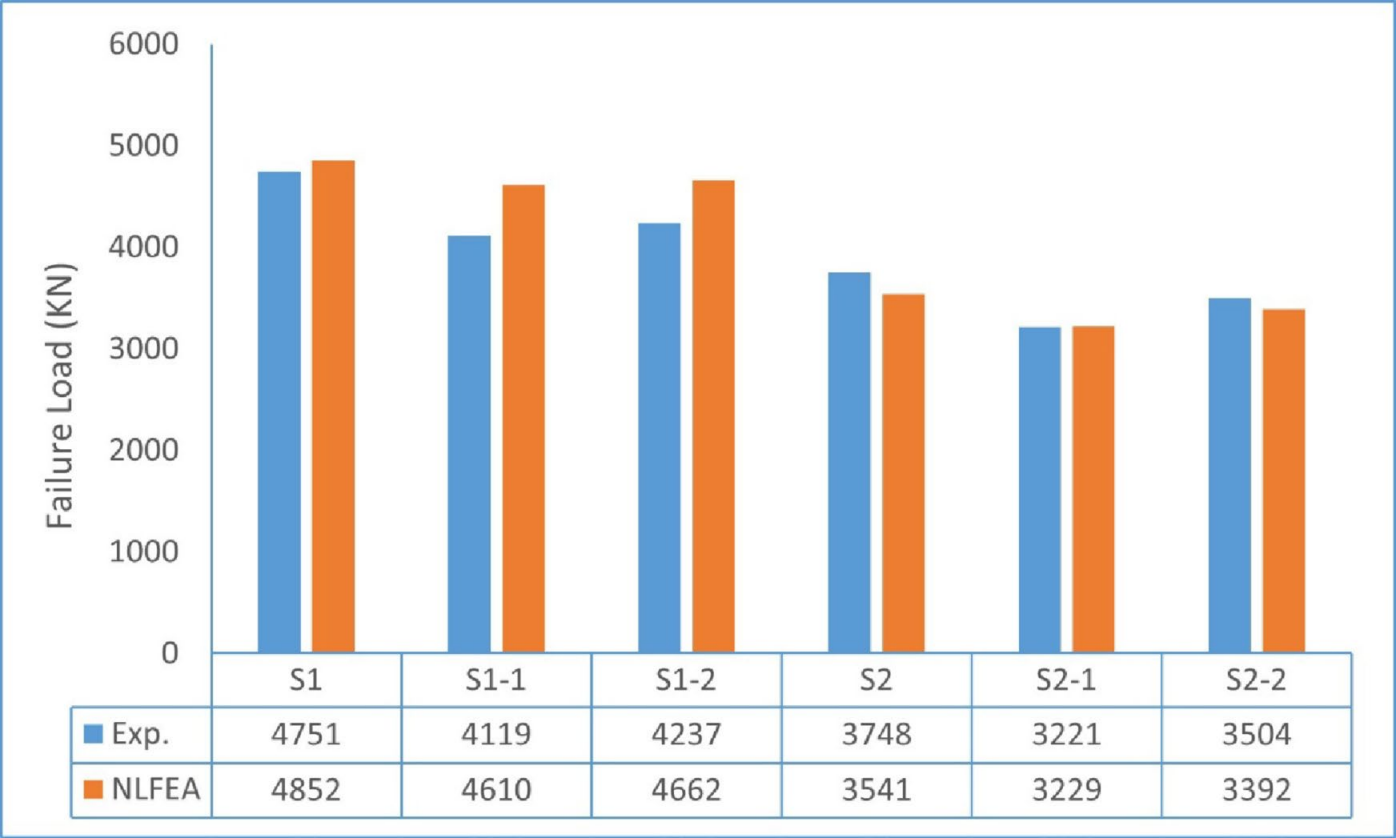
Comparison of ultimate failure loads of tested wall specimens using Experimental and NLFEA.

The SDEG is presented in Fig. 31 as the scalar stiffness degradation parameter obtained from the concrete damage plasticity (CDP) model in ABAQUS, and is represented by values between 0 (undamaged material) and 1 (fully damaged material). The notation “Avg: 75%” shown in the contour legend refers to the averaging threshold used during post-processing in ABAQUS to smooth contour results, and does not correspond to a specific damage level (Figs. 32,33).

### Comparison between experimental and NLFEA results

There was a good agreement between experimental and NLFEA ultimate failure loads. The numerical predictions were very similar to the experimental capacities for all tested specimens, with discrepancies ranging from approximately 0.25% to 11.9%. Moreover, the numerical results were consistent with the experimental trends regarding the load–deformation response, stiffness degradation, and crack distribution under both concentric and eccentric loading conditions. This agreement confirms the adequacy and acceptable predictive accuracy of the adopted NLFEA model.

**Error Analysis between Experimental and FE Predictions**.

A quantitative comparison was performed between the experimental ultimate loads (Pu) and those predicted by the nonlinear FE analysis. The percentage deviation was calculated as:

$$100\times\frac{\mid FE-EXP\mid}{EXP}=(\%)Deviation$$

As shown in Table 14, the deviation ranged between 0.24% and 11.91%, confirming that the FE model provided acceptable agreement with the experimental response.

**Table 14:** Error analysis between experimental and FE predicted ultimate loads (Pu).

Specimen	Pu (EXP) (kN)	Pu (FE) (kN)	Deviation (%)
**S1**	4751	4852.15	2.13%
**S1-1**	4119	4609.70	11.91%
**S1-2**	4237	4661.50	10.02%
**S2**	3748	3541.42	5.51%
**S2-1**	3221	3228.63	0.24%
**S2-2**	3504	3392.35	3.19%

### Comparison with previous studies

The behavior observed in this study is generally consistent with that reported in earlier research on FRP-reinforced compression members. Previous studies have shown that GFRP-reinforced elements typically exhibit lower axial load capacity than steel-reinforced ones, mainly due to the lower elastic modulus of GFRP and the absence of yielding.

The distributed pattern of cracking seen and relatively strong post-cracking response were consistent with those reported in the previous literature on FRP-reinforced concrete members. Nevertheless, the majority of previous works have concentrated primarily on beams and columns with relatively little focus on reinforced concrete wall members exhibiting concentric and eccentric loading conditions.

Against this background, the current study adds to the literature by presenting combined experimental and numerical insights into the behavior of GFRP-reinforced concrete walls under different loading scenarios.

## Conclusions

Based on the experimental study and nonlinear finite element analysis of RC walls reinforced using steel and GFRP bars under concentric and eccentric axial loading, the following conclusions may be drawn:

1. The study provides an empirical evaluation of the axial behavior of GFRP-reinforced RC walls. Reinforcement type influences strength, stiffness, and failure characteristics. GFRP-reinforced walls exhibit more distributed cracking patterns compared to steel-reinforced specimens. Although cracking initiates at lower load levels, the post-cracking response remains stable without sudden brittle failure under the conditions tested.
2. The use of GFRP bars reduces ultimate axial capacity by approximately 10.8–13.3% under concentric load and 6.5–14.1% under eccentric load. This reduction, however, falls within a range that may be acceptable for certain structural applications, particularly where corrosion resistance is prioritized over maximum capacity.
3. Load eccentricity substantially affects structural response. Increased eccentricity reduces stiffness and produces greater deformation. This effect is more significant than the influence of reinforcement type on serviceability behavior within the present experimental scope.
4. Despite the linear-elastic behavior of GFRP material, the tested walls demonstrate reasonable ductility and deformation capacity. This behavior is likely attributable to concrete cracking processes and stress redistribution mechanisms, though further validation across different configurations remains necessary.
5. The finite element model developed using the concrete damaged plasticity (CDP) approach in ABAQUS shows good agreement with experimental results regarding load capacity, stiffness, and failure patterns. This agreement is valid within the parameter ranges investigated.
6. The findings suggest that GFRP bars may serve as corrosion-resistant alternatives to steel reinforcement in RC wall systems. This potential is most relevant for aggressive environments where durability is a critical concern, provided that the associated reduction in load capacity is deemed acceptable for the intended application.
7. Current design provisions, specifically ACI 440.1R, CSA S806, and Eurocode 2, exhibit certain limitations when applied to GFRP-reinforced walls. The results presented herein offer preliminary practical guidance for design in aggressive environments, although additional experimental and analytical validation is strongly recommended before broad adoption.

## Study limitations

**1. Specific wall thickness and concrete strength (150 mm and ≈ 60 MPa**,** respectively).**

The results are only valid for that exact geometry and high-strength concrete. Thinner/wider tubes or lower/higher concrete strengths might behave differently (e.g., different confinement effects, buckling modes).

**2. Only monotonic axial loading was considered.**

Real-world structures experience cyclic loads (e.g., earthquakes, wind, vibrations), fatigue, or eccentric loading. Monotonic tests show ultimate capacity but not degradation, stiffness change, or ductility under repeated loading.

**3. Neither the environmental effect nor long-term behavior was considered.**

Factors like freeze–thaw, corrosion, creep, shrinkage, and temperature effects are ignored. Long-term durability and serviceability (e.g., deflection over time) remain unknown.

**4. Bond–slip behavior was not modeled explicitly.**

Composite action between steel and concrete depends on the bond. Without modeling bond–slip, the study may overestimate stiffness or strength, and cannot predict slip-induced failure or load transfer length.

## Recommendations for future research

**1. Explore cyclic and long-term behavior**.

Future research should investigate these behaviors to make the findings applicable to actual construction.

**2. Extend testing to larger-scale specimens**.

Future work needs to test larger scales to confirm whether the results remain valid.

**3. Investigate hybrid reinforcement systems**.

Future research should explore these hybrid systems to enhance ductility, crack control, and structural strength.

**4. Improve numerical modeling by including bond–slip and progressive damage**.

Future models should include bond–slip behavior and progressive damage mechanisms, such as concrete cracking, steel yielding, and debonding to produce more realistic predictions.

## Data Availability

All data generated or analyzed during this study are included in this published article.
